# Requirement of microtubules for secretion of a micronemal protein CpTSP4 in the invasive stage of the apicomplexan *Cryptosporidium parvum*

**DOI:** 10.1128/mbio.03158-23

**Published:** 2024-01-24

**Authors:** Dongqiang Wang, Peng Jiang, Xiaodong Wu, Ying Zhang, Chenchen Wang, Meng Li, Mingxiao Liu, Jigang Yin, Guan Zhu

**Affiliations:** 1 State Key Laboratory for Diagnosis and Treatment of Severe Zoonotic Infectious Diseases, Key Laboratory for Zoonosis Research of the Ministry of Education, Institute of Zoonosis, and College of Veterinary Medicine, Jilin University, Changchun, China; Albert Einstein College of Medicine, New York, New York, USA

**Keywords:** protozoa, apicomplexan, *Cryptosporidium*, microneme, TSP-domain containing protein, secretion, microtubule, intracellular trafficking, heparin-binding domain/motif

## Abstract

The zoonotic *Cryptosporidium parvum* is a global contributor to infantile diarrheal diseases and opportunistic infections in immunocompromised or weakened individuals. Like other apicomplexans, it possesses several specialized secretory organelles, including micronemes, rhoptry, and dense granules. However, the understanding of cryptosporidial micronemal composition and secretory pathway remains limited. Here, we report a new micronemal protein in *C. parvum*, namely, thrombospondin (TSP)-repeat domain-containing protein-4 (CpTSP4), providing insights into these ambiguities. Immunostaining and enzyme-linked assays show that CpTSP4 is prestored in the micronemes of unexcysted sporozoites but secreted during sporozoite excystation, gliding, and invasion. In excysted sporozoites, CpTSP4 is also distributed on the two central microtubules unique to *Cryptosporidium*. The secretion and microtubular distribution could be completely blocked by the selective kinesin-5 inhibitors SB-743921 and SB-715992, resulting in the accumulation of CpTSP4 in micronemes. These support the kinesin-dependent microtubular trafficking of CpTSP4 for secretion. We also localize γ-tubulin, consistent with kinesin-dependent anterograde trafficking. Additionally, recombinant CpTSP4 displays nanomolar binding affinity to the host cell surface, for which heparin acts as one of the host ligands. A novel heparin-binding motif is identified and validated biochemically for its contribution to the adhesive property of CpTSP4 by peptide competition assays and site-directed mutagenesis. These findings shed light on the mechanisms of intracellular trafficking and secretion of a cryptosporidial micronemal protein and the interaction of a TSP-family protein with host cells.

**IMPORTANCE:**

*Cryptosporidium parvum* is a globally distributed apicomplexan parasite infecting humans and/or animals. Like other apicomplexans, it possesses specialized secretory organelles in the zoites, in which micronemes discharge molecules to facilitate the movement and invasion of zoites. Although past and recent studies have identified several proteins in cryptosporidial micronemes, our understanding of the composition, secretory pathways, and domain-ligand interactions of micronemal proteins remains limited. This study identifies a new micronemal protein, namely, CpTSP4, that is discharged during excystation, gliding, and invasion of *C. parvum* sporozoites. The CpTSP4 secretion depends on the intracellular trafficking on the two *Cryptosporidium*-unique microtubes that could be blocked by kinesin-5/Eg5 inhibitors. Additionally, a novel heparin-binding motif is identified and biochemically validated, which contributes to the nanomolar binding affinity of CpTSP4 to host cells. These findings indicate that kinesin-dependent microtubular trafficking is critical to CpTSP4 secretion, and heparin/heparan sulfate is one of the ligands for this micronemal protein.

## INTRODUCTION

The zoonotic *Cryptosporidium parvum* is one of the leading causes of moderate to deadly diarrheal diseases in infants and opportunistic infections in immunocompromised patients (1–3). Like other apicomplexans (e.g., *Toxoplasma* and *Plasmodium*), cryptosporidial zoites possess several specialized secretory organelles, namely, micronemes, rhoptries, and dense granules (4, 5). Among them, micronemes are distributed in the sporozoite anterior and secrete various adhesins and peptidases to facilitate the moving zoites to cross the mucus layer and interact with host cells (5–8). Despite their importance in parasite invasion, only a limited number of micronemal proteins have been validated in *Cryptosporidium* (9–11). Studies on the secretory pathways of cryptosporidial micronemal proteins and their domain-ligand interactions are also limited.

Based on studies on *Toxoplasma* and *Plasmodium*, microneme secretion is triggered by a signaling cascade involving intracellular cyclic nucleotides, calcium levels, phosphatidic acid, and downstream effectors (6, 8). Micronemal proteins are thought to be secreted via membrane fusion of secretory vesicles with parasite plasma membranes, for which only a handful of molecular players have been identified (8, 12, 13). Cytoskeletons, including microtubules and F-actin, are known to participate in intracellular trafficking of vesicles in the secretory pathways (14, 15). *Toxoplasma* and *Plasmodium* possess an array of cortical and subpellicular microtubules (SPMTs) for maintaining the shape of zoites (16), while currently there is no evidence that SPMTs are involved in intracellular trafficking of secretory molecules. The two short intraconoidal microtubules, unseen in *Cryptosporidium*, are implied for transporting “microtubule-associated vesicles” to the apical tip during the rhoptry docking and exocytosis (6, 8, 11). F-actin, which is part of the glideosome in gliding zoites and the moving junction formed during the parasite invasion, is implied in the apical positioning of rhoptries (17). Conversely, *C. parvum* lacks SPMTs but possesses two central microtubules extended from the apex to the central and posterior regions of the zoites, respectively (18). The two central microtubules appear unique to *Cryptosporidium*, but their biological roles remain unclear.

Here, we report the cellular and biochemical characterizations of a *C. parvum* thrombospondin (TSP)-repeat domain-containing protein (aka thrombospondin-related adhesive/anonymous protein), namely, CpTSP4. CpTSP4 is prestored in the micronemes of the sporozoites but secreted during excystation, gliding motility, and invasion of the sporozoites. During secretion, CpTSP4 is transported on the two central microtubules in sporozoites. Secretion and microtubular trafficking could be fully blocked by selective kinesin-5 inhibitors. Additionally, CpTSP4 possesses a novel heparin-binding motif (HBM) that contributes to its nanomolar binding affinity to host cells. These studies reveal a biological function for the cryptosporidial-unique microtubules in the intracellular trafficking of a micronemal protein and uncover one of the host cell ligands for an apicomplexan TSP-family adhesive protein.

## RESULTS

### CpTSP4 is prestored in micronemes but transported along the two central microtubules for secretion in sporozoites during and after excystation

**Fig 1:**
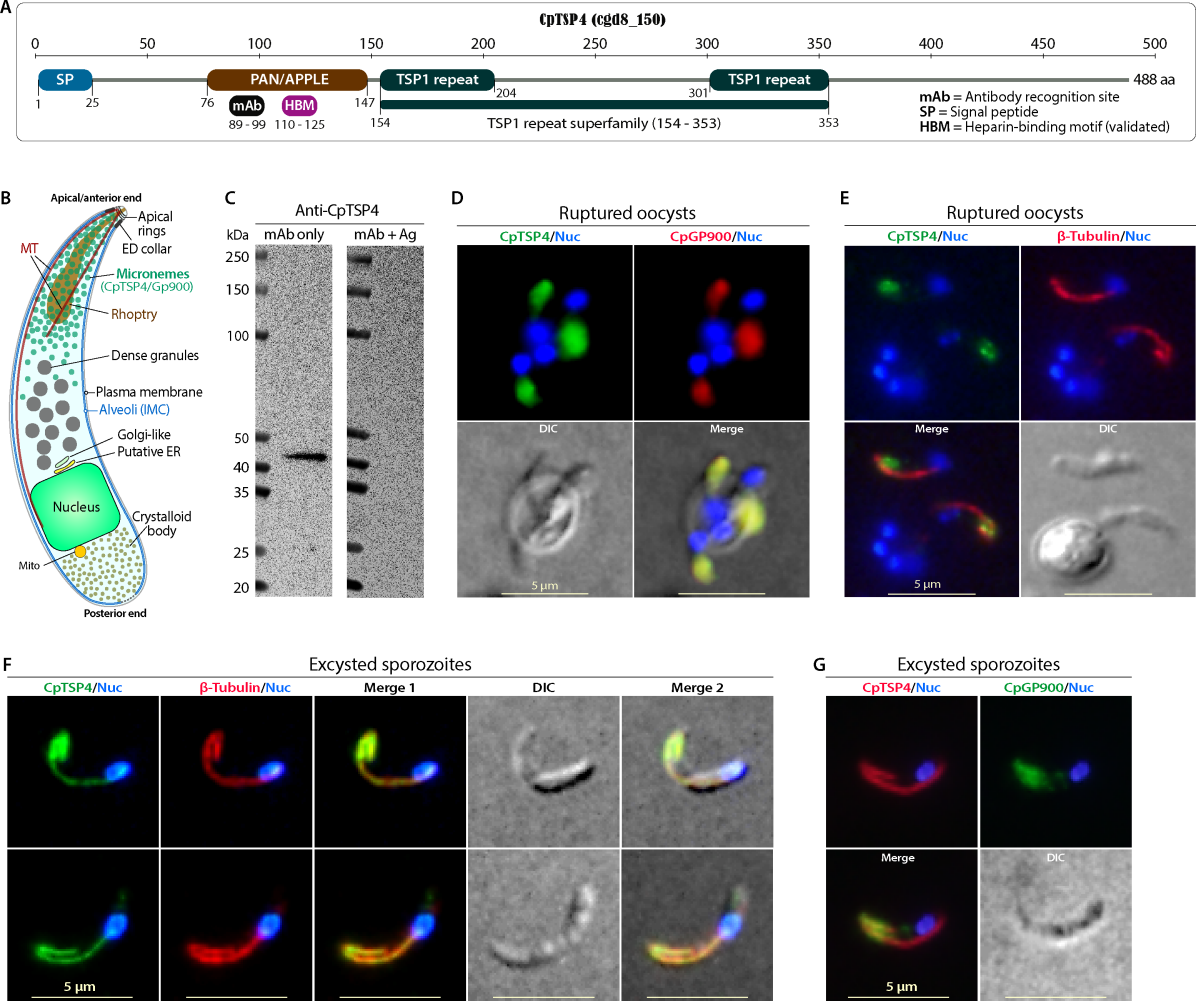
Distribution of CpTSP4 in the micronemes and on the microtubules in *C. parvum* sporozoites. (**A**) Architecture of CpTSP4 (cdg8_150; 488 aa), showing an N-terminal SP, a PAN/APPLE domain, and two TSP1 repeats. The site recognized by the anti-CpTSP4 monoclonal antibody (mAb) and the HBM determined in this study are indicated. (**B**) Illustration of the major structures in a *C. parvum* sporozoite that may be frequently discussed in this study, including micronemes and two unique central microtubules. ED collar, electron-dense collar; IMC, inner membrane complex; MT, microtubule. (**C**) Western blot analysis using anti-CpTSP4 mAb detected a single band from the sporozoite crude extract (marked with “mAb only”). The band was eliminated by pre-incubating the mAb with peptide antigen (marked with “mAb + Ag”). (D–G) Dual-labeling IFA of CpTSP4 with CpGP900 (a microneme marker) or microtubules in unexcysted (but ruptured) oocysts or excysted sporozoites. In unexcysted or ruptured oocysts, CpTSP4 was concentrated in the apical region. It was colocalized with CpGP900 (**D**), but not with microtubules (**E**). In excysted sporozoites, CpTSP4 started to distribute over the two central microtubules (**F**), differing from the mainly apically distributed CpGP900 (**G**). This IFA used the following antibodies: an anti-CpTSP4 mAb, a rabbit anti-CpGP900 C-terminal epitope pAb, and a rabbit anti-CpTubB pAb. Intact oocysts were ruptured in fixative by three freeze-and-thaw cycles to allow access to antibodies to sporozoites. DIC, differential interference contrast microscopy; Nuc, nuclei counter-stained with 4,6-diamidino-2-phenylindole.

The *C. parvum* genome encodes 12 TSP-family proteins (CpTSP1 to 12) (see a list of CpTSP proteins in Table S1 in the supplemental material) (19, 20). Among them, CpTSP1 (aka TRAP-C1) and CpTSP8 were localized to the apical region and/or surface of excysted sporozoites (20–22). This study focused on CpTSP4 (Gene ID: cgd8_150; Genbank: XP_625479) based on its moderate size (488 aa) and relatively simple domain architecture, i.e., an N-terminal signal peptide (SP; 25 aa), a single PAN/APPLE domain, and two TSP1-like repeats, but lacking a transmembrane domain (TMD) (Fig. 1A). To determine the subcellular location of CpTSP4 in the parasite (see Fig. 1B for illustration of sporozoite), an anti-CpTSP4 monoclonal antibody (mAb) was raised in-house against a short epitope (^89^KIKKADSWQEC^99^). A single band from sporozoite crude extract was recognized by this mAb in western blot analysis, in which the signals could be eliminated by pre-incubation with the peptide immunogen (Fig. 1C), supporting the mAb’s specificity. In intact/unexcysted oocysts that were ruptured by freeze-and-thaw to allow access of antibody to sporozoites, anti-CpTSP4 mAb labeled the anterior region of sporozoites by immunofluorescence assay (IFA) that could be colocalized with a rabbit polyclonal antibody (pAb) against CpGP900—a known microneme marker (Fig. 1D; see Fig. S1 for additional images) (23, 24). The staining pattern differs from that of the filamentous microtubules labeled by an affinity-purified rabbit pAb on *C. parvum* β-tubulin (CpTubB) (Fig. 1E) (18). These observations indicate that CpTSP4 is prestored in the micronemes in sporozoites before excystation.

Upon excystation, some CpTSP4 molecules started to show distribution along with the two central microtubules that could be fully colocalized with anti-CpTubB pAb (Fig. 1F; see Fig. S2 for additional images using secondary antibodies with altered green and red fluorophores), implying intracellular trafficking of CpTSP4 on the two microtubules. These microtubules were recently reported to be *Cryptosporidium* unique (18), differing from SPMTs in *Toxoplasma* and *Plasmodium* (4, 25). The labeling of microtubules by the anti-CpTSP4 mAb was not caused by its cross-reaction with *C. parvum* tubulin, because: (i) the mAb did not label the microtubules in unexcysted sporozoites (Fig. 1E); and (ii) when anti-CpTSP4 mAb and anti-CpTubB pAb were individually pre-incubated with their peptide immunogens, each immunogen only eliminated fluorescent signals from the corresponding antibody but not those from the other antibody (see Fig. S3 in the supplemental material). For comparison, anti-CpGP900 pAb mainly labeled the micronemes in the anterior of sporozoites, showing no filamentous distribution (Fig. 1G). The labeling pattern of CpGP900 in excysted sporozoites is consistent with our previous study (23), implying that CpTSP4 and CpGP900 use different pathways for secretion.

**Fig 2:**
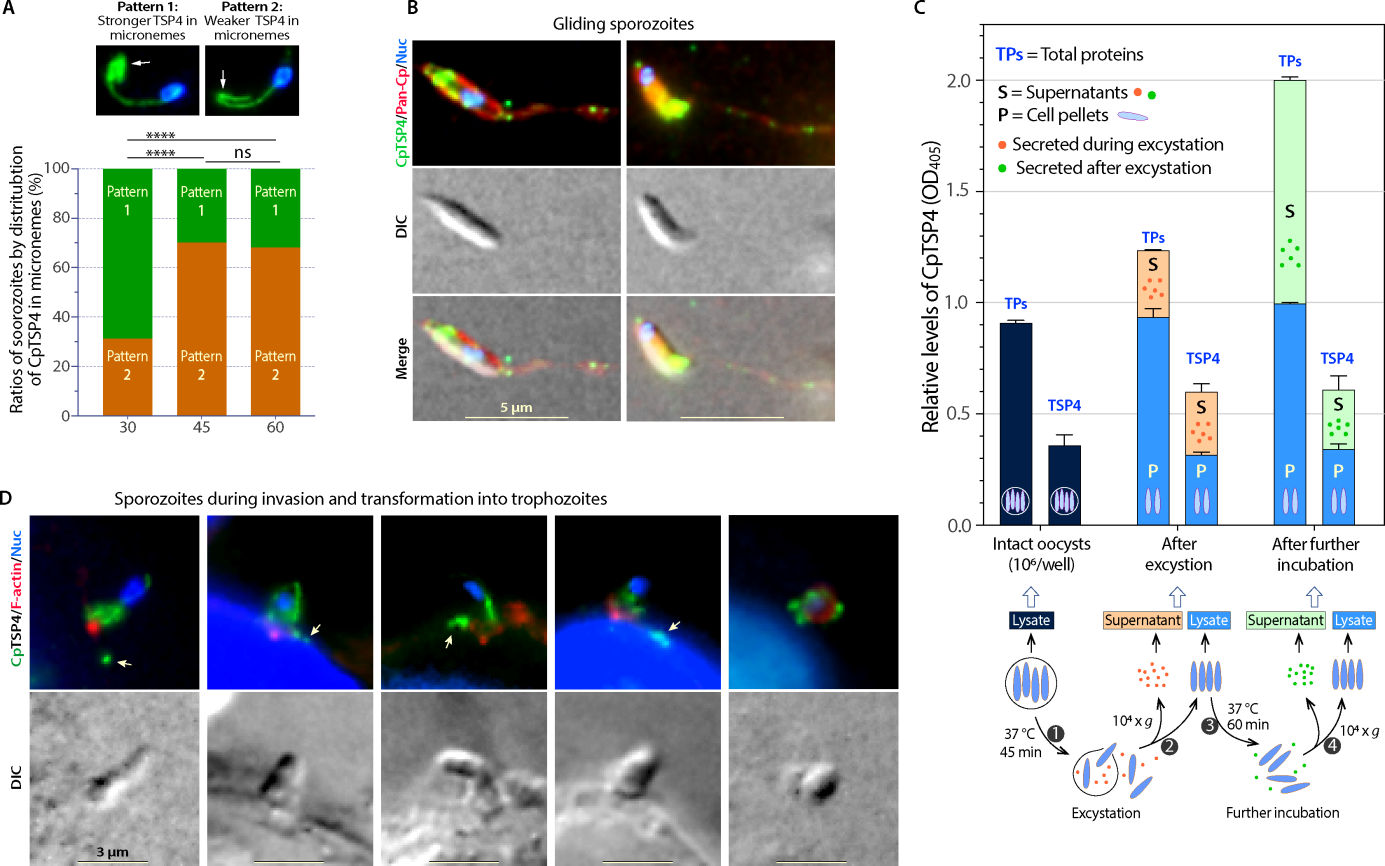
Secretion of CpTSP4 from *C. parvum* sporozoites during and after excystation and during invasion, as detected by immunostaining using anti-CpTSP4 mAb. (**A**) During the course of excystation, the ratios of sporozoites showing strong CpTSP4 signals in the anterior region (pattern 1) were reduced, whereas those showing weak anterior CpTSP4 signals (pattern 2) were increased (*N* > 200 at each time point). Note: The two sporozoite images from Fig. 1F (left panel) are reused here for easier comparison of patterns 1 and 2 sporozoites. ****P < 0.0001 by two-sided Fisher’s exact test; ns, no significance. (**B**) Immunostaining of CpTSP4 in free sporozoites allowed to glide on poly-L-lysine-coated slides in RPMI-1640 medium for 20 min at 37°C. Discharged CpTSP4 was visible in granular form (green) over the gliding trails (red) and co-stained with a rabbit pAb against total sporozoite proteins (marked as Pan-Cp). (**C**) Semi-quantitative ELISA detection of secreted CpTSP4 in the supernatants (**S**) and non-secreted CpTSP4 in the parasite cell pellets (**P**) collected immediately after excystation or after further incubation of excysted sporozoites in fresh RPMI-1640 medium. Intact oocysts were used as loading controls. Lysates and supernatants were coated in microplates and detected with anti-CpTSP4 mAb. The rabbit pAb against total sporozoites proteins (TPs) was used in parallel for reference and positive control. Each sample contained 10^6^ oocysts or equivalent (*N* = 3/sample). Bars showed the standard error of the mean (SEM). (**D**) Immunostaining of CpTSP4 (green) in sporozoites during the invasion and transformation into trophozoites. Host cell F-actin was stained with rhodamine-phalloidin (red). Arrows indicate CpTSP4 discharged into extracellular space. DIC, differential interference contrast microscopy; Nuc, nuclei counter-stained with 4,6-diamidino-2-phenylindole.

The secretion of CpTSP4 resulted in the reduction of CpTSP4 in the anterior of the sporozoites during excystation, while in the meantime, the signals over the two microtubules remained relatively constant (Fig. 2A). Secreted CpTSP4 molecules were detected by IFA along the sporozoite gliding trails, mostly in granular form (Fig. 2B; see Fig. S4 for additional images). Semi-quantitative enzyme-linked immunosorbent assay (ELISA) detected CpTSP4 from the supernatants collected after excystation (indicating secretion during excystation) and the supernatants when excysted sporozoites were further incubated (indicating secretion after excystation) (Fig. 2C). The contents of intracellular CpTSP4 in unexcysted oocysts, excysted sporozoites, and further incubated sporozoites remained relatively constant. For comparison, a pAb against parasite total proteins detected similar patterns of secreted proteins (Fig. 2C). The data indicate that CpTSP4 is continuously secreted, and new CpTSP4 molecules are synthesized during or after excystation. During the sporozoite invasion of host cells, CpTSP4 is continuously distributed over the shortening microtubules in the transforming sporozoites and discharged to the extracellular space (Fig. 2D).

**Fig 3:**
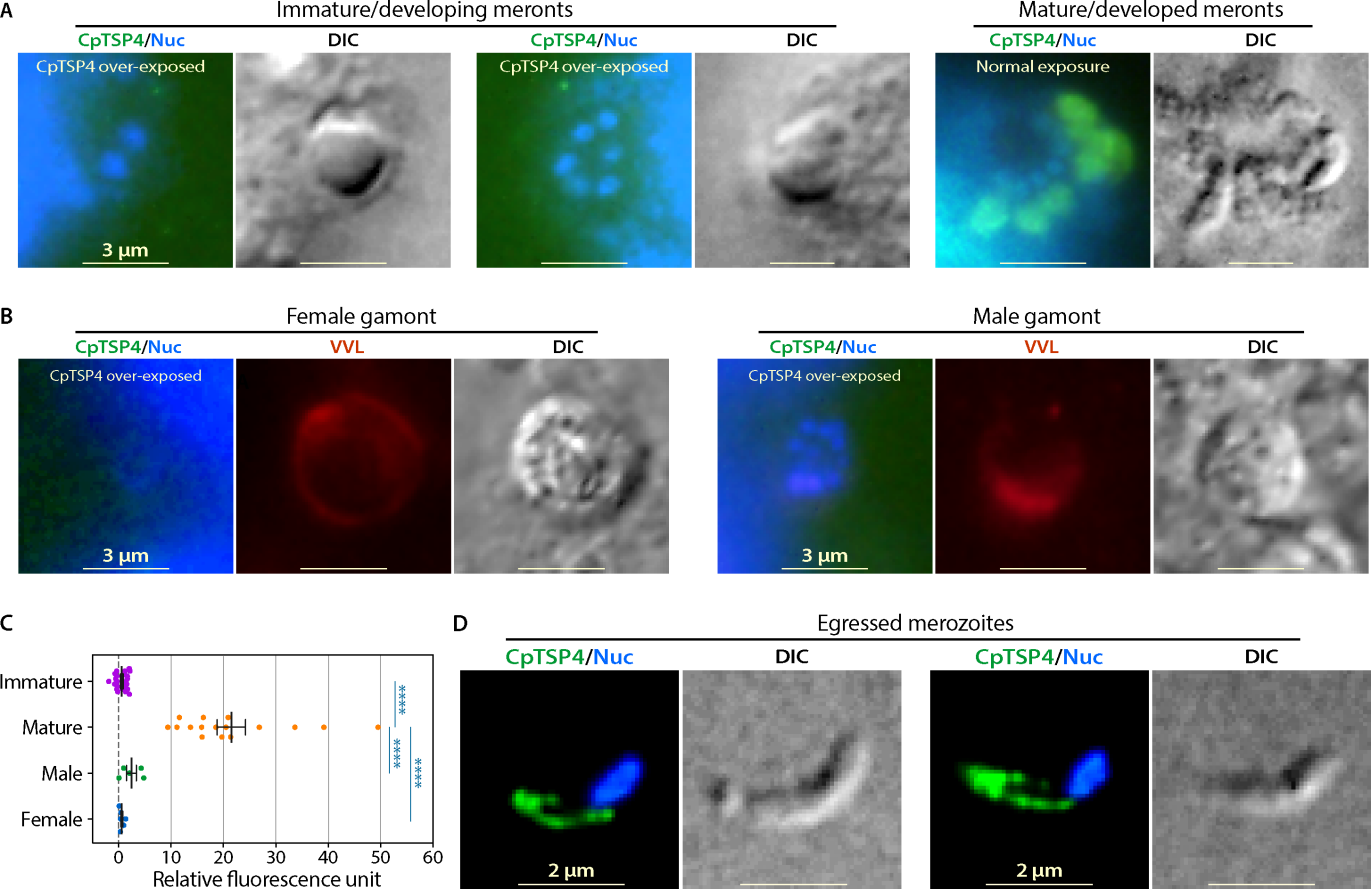
Distribution of CpTSP4 in the intracellular stages and egressed merozoites of *C. parvum* by immunostaining with an anti-CpTSP4 mAb. (**A**) In asexual stages, CpTSP4 was absent in immature meronts that are typically morphologically smooth and contain only two to six nuclei (green; only background signals were observed even in over-exposed images), but present in mature meronts that are morphologically rough by containing fully developed merozoites. A mature meront contained eight nuclei, but some nuclei were outside of the focal point. (**B**) In sexual stages, in which *C. parvum* were outlined by co-staining with *Vicia villosa* lectin (VVL)-Texas Red, CpTSP4 (green) was absent in both female [one large nucleus with no or weak 4,6-diamidino-2-phenylindole (DAPI)-staining] and male (>8 small nuclei) gamonts/gametes. In the green channel, only background signals were observed, even in over-exposed images. (**C**) Scatter plot of the relative fluorescence intensities derived from anti-CpTSP4 mAb in *C. parvum* asexual and sexual stages. In the measurement, signals from parasite cells were subtracted from the background signals of the same size collected from nearby areas. Again, strong CpTSP4 signals were detected in mature meronts, whereas only near-background signals were detected in immature meronts and male/female gamonts. ****P < 0.0001 by the Tukey multiple comparison test. (**D**) Immunostaining of CpTSP4 in egressed merozoites shows a similar distribution as seen in excysted sporozoites. DIC, differential interference contrast microscopy; Nuc, nuclei counter-stained with DAPI.

In intracellular stages, CpTSP4 is absent in immature/developing meronts (2–6 nuclei), but present in mature/developed meronts containing developed merozoites (eight nuclei) (Fig. 3A and C; also see Fig. S5 for images of meronts containing various numbers of nuclei). The distribution on one side of the nucleus in the packed meronts is similar to CpGP900, which is also present only in fully developed meronts (23). Like CpGP900, CpTSP4 was absent in male and female gametes (Fig. 3B and C). In egressed merozoites, CpTSP4 was also localized to the anterior region and the two microtubules (Fig. 3D; see Fig. S6 for additional images), suggesting that CpTSP4 plays roles shared by sporozoites and merozoites. Collectively, CpTSP4 is a micronemal protein that exists only in parasitic zoites. It is prestored in micronemes in unexcysted oocysts but transported over the two central microtubules for secretion during excystation, gliding, and invasion. It is worth noting that, in agreement with the lack of TMD, CpTSP4 is absent on the sporozoite plasma membranes, suggesting that CpTSP4 is not attached to sporozoites via glycosylphosphatidylinositol anchor or binding to other surface proteins in the parasite.

### Selective kinesin-5/Eg5 inhibitors (SB-743921 and SB-715992) could completely block the secretion of CpTSP4 and its transport on the central microtubules

**Fig 4:**
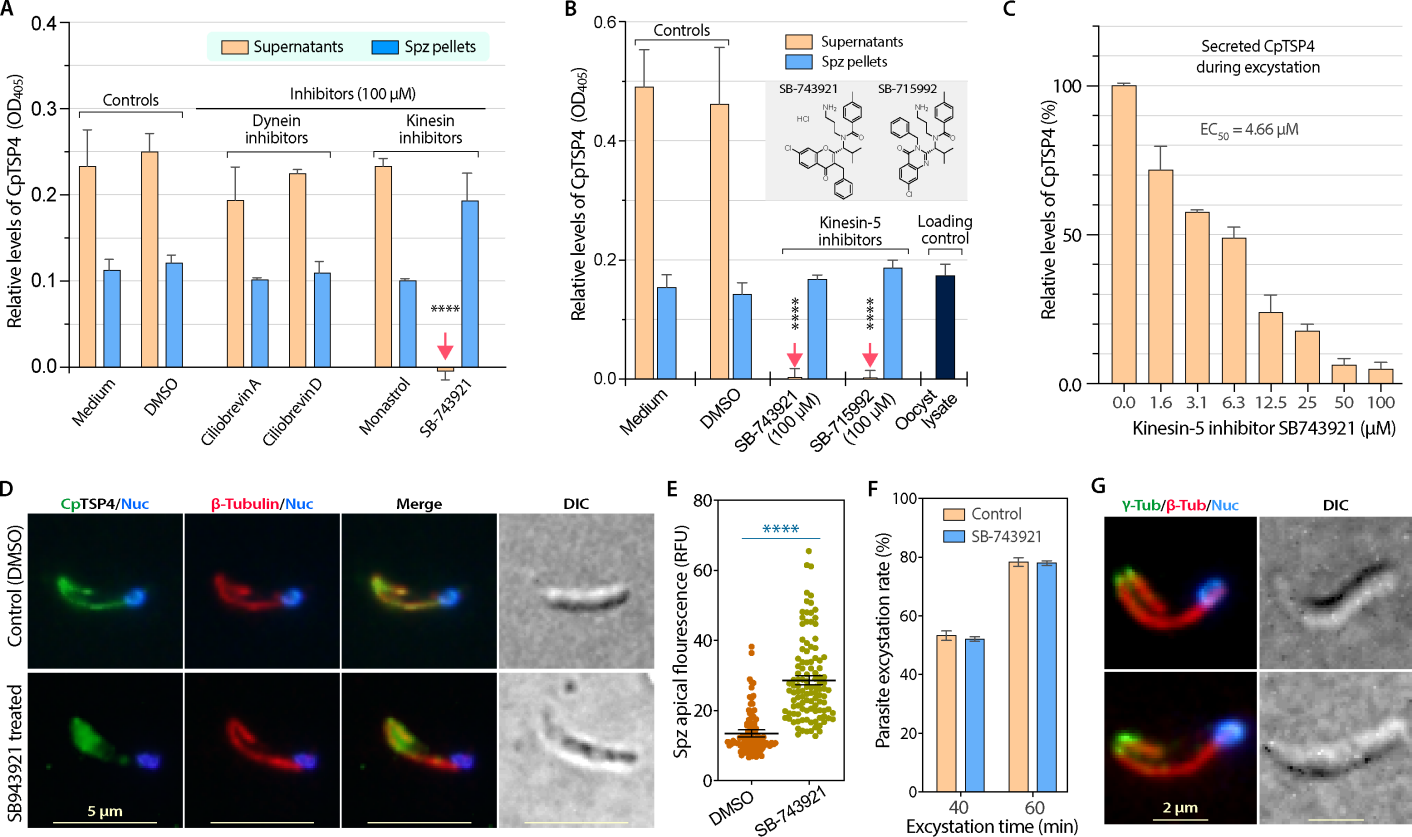
Effect of the selective kinesin-5 inhibitors SB-743921 and SB-715992 on the secretion of CpTSP4 and distribution in sporozoites. (**A**) Effects of selected dynein and kinesin inhibitors on the secretion of CpTSP4 from *C. parvum* during excystation. Oocysts (10^6^ per sample) were incubated in excystation medium with or without inhibitors (100 µM) for 1 h. Supernatants and lysates of sporozoite pellets (Spz) were coated in microplates. CpTSP4 was detected by semi-quantitative ELISA using an anti-CpTSP4 mAb. The selective kinesin-5 inhibitor SB-743921 completely inhibited the secretion of CpTSP4 (indicated by red arrows). The small negative reads in the SB-743921-treated group (OD_405_ = −0.005) were a result of subtraction of background (vs blank wells coated with medium only). (**B**) Complete inhibition of a second kinesin-5 inhibitor SB-715992 in comparison with SB-743921 (both at 100 µM; inset depicts their chemical structures) on the secretion of CpTSP4 from the parasite after excystation for 1 h by ELISA (10^6^ oocysts/sample). (**C**) Dose-dependent inhibition of SB-743921 on the secretion of CpTSP4 from the parasite after excystation for 1 h by ELISA (10^6^ oocysts/sample). (D–F) Treatment of SB-743921 (50 µM for 1 h) resulted in the accumulation of CpTSP4 in the anterior region of excysted sporozoites and loss of its distribution on the two microtubules, as shown by dual-labeling IFA (**D**) and by measuring the relative fluorescence intensities in the sporozoite apical regions (**E**). The same treatment had no apparent effect on the parasite morphology (**D**) or on the parasite excystation rate (**F**). (**G**) Co-localization IFA of microtubules (red) using anti-CpTubB pAb with γ-tubulin (green) using a commercial anti-γ-tubulin mAb. Error bars show the standard error of the mean (SEM; *N* = 3 in bar charts or >100 in the scatter plot). ****P < 0.0001 based on two-way ANOVA with Tukey’s multiple comparisons (**A and B**) or Student’s *t*-test (**E**).

Dynein and kinesin are the two molecular motors involved in microtubule trafficking (15, 26). To determine which motor was involved in the trafficking of CpTSP4, two dynein inhibitors and two kinesin inhibitors (100 µM) were first tested for effects on the secretion of CpTSP4. Inhibitors were present throughout the entire course of excystation (37°C for 1 h) to ensure immediate contact with the excysting sporozoites. Strikingly, the CpTSP4 secretion from sporozoites was completely suppressed by SB-743921 (CAS 940929-33-9), a highly selective inhibitor of human kinesin-5 (aka Eg5, or kinesin spindle protein) (27, 28), but not by the other three inhibitors (Fig. 4A). The secretion of CpTSP4 from sporozoites could also be blocked by another kinesin-5/Eg5 inhibitor, SB-715992 (CAS 336113-53-2; an SB-743921 analog) (Fig. 4B). SB-743921 inhibited CpTSP4 secretion in a dose-dependent manner, showing low micromolar inhibitory activity (*EC*_50_ = 4.66 µM) (Fig. 4C). The inhibition of CpTSP4 secretion and transport on microtubules by SB-743921 was further confirmed by IFA (Fig. 4D and E; see Fig. S7 for additional images), in which the presence of SB-743921 during excystation (50 µM for 1 h) resulted in the accumulation of CpTSP4 in the anterior region of the sporozoites and the loss of CpTSP4 signals from the two microtubules. Under the same condition, SB-743921 had no apparent effect on the parasite morphology (Fig. 4D) or excystation rate (Fig. 4F).

These observations suggest the essentiality of kinesin-dependent microtubule trafficking in the secretion of CpTSP4. The kinesin responsible for the trafficking of CpTSP4 cargoes would share certain homology with human kinesin-5/Eg5 at the motor domain (MD), the binding site for SB-743921/SB-715992. Indeed, the *C. parvum* genome encodes seven kinesin orthologs (29), of which the kinesin-5 ortholog (cgd6_4210; designated as CpKin5) is highly unique among those in *Cryptosporidium* and other apicomplexans. CpKin5-MD shares much higher homology to human kinesin-5/Eg5 (73.3% similarity score) than to the other six *C. parvum* kinesins (31.3% and 46.3% similarities), or to the kinesin-5 orthologs from *Toxoplasma* and *Plasmodium* (49.9% and 54.4%) (see Table S2 and S3 for similarity/identity scores and Fig. S8 for sequence comparison). Molecular docking also shows that CpKin5 and human kinesin-5/Eg5 share a conserved SB-743921-binding pocket (see Fig. S9 for the structural model) (28). While more experimental evidence is needed, the current data strongly suggest that CpKin5 is the likely molecular motor involved in the microtubular transport of CpTSP4.

The involvement of kinesin implies an anterograde transport of CpTSP4-cargoes towards the posterior end on the two central microtubules (i.e., from the minus to the plus end), in which the minus end is typically attached to the microtubule-organizing center (MTOC). For *Toxoplasma* and *Plasmodium* SPMTs, apical polar rings serve as unconventional MTOCs (4). However, we detected γ-tubulin at the anterior ends of the two *C. parvum* microtubules (Fig. 4F), suggesting that the parasite might use γ-tubulin-based conventional MTOC for these microtubules (30). The polarity of the two central microtubules agrees with the function of kinesins that slide towards the plus end of microtubules for transporting CpTSP4-cargoes from the anterior micronemes.

### A novel heparin-binding motif contributes to approximately half of the host cell-binding activity of CpTSP4

**Fig 5:**
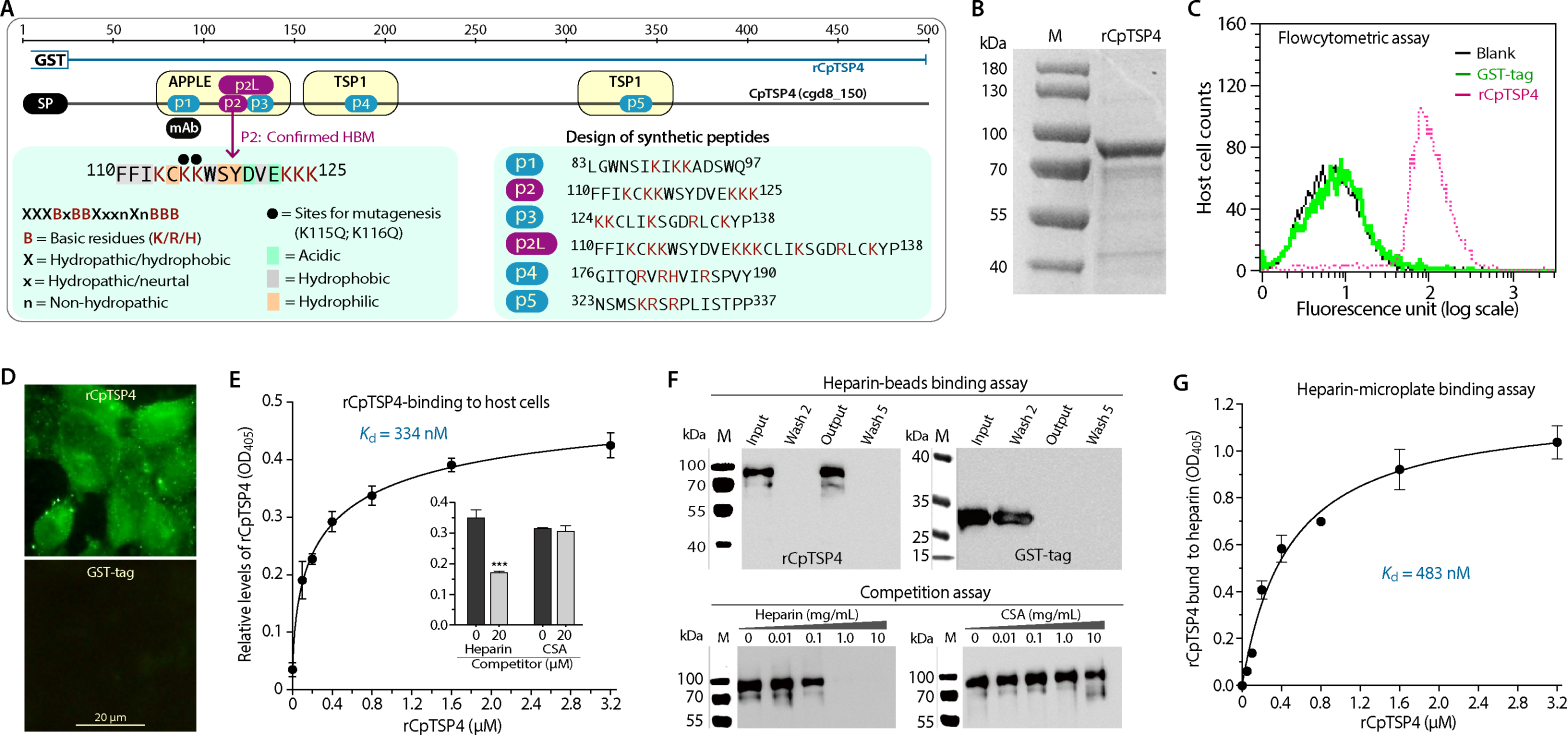
Specific binding of GST-fused recombinant CpTSP4 (rCpTSP4) to host cells and to heparin. (**A**) Illustration of rCpTSP4 and the positions of candidate HBM (sites and sequences) for designing synthetic peptides (p1 to p5), including the two amino acids at the p2 site subjected to site-directed mutagenesis (K115Q/K116Q). The purple color indicates the novel HBM site determined in this study. (**B**) Purified rCpTSP4 in SDS-PAGE gel. (C–D) Binding of rCpTSP4 (vs GST-tag control) to host cells as determined by flow cytometry (**C**) and immunofluorescence assay (**D**). (**E**) Binding kinetics of rCpTSP4 to formalin-fixed HCT-8 cells by enzyme-linked assay (App. *K*_d_ = 334 nM). The inset shows that the binding of rCpTSP4 (2.0 µM) to HCT-8 cells could be reduced by heparin (20 µM) but not by chondroitin sulfate A (CSA; 20 µM). ***P < 0.001 by a two-tailed Student’s *t*-test. (**F**) Binding of rCpTSP4 to heparin-agarose beads (vs GST-tag; upper panel) and competition of the binding by heparin (vs CSA; lower panel). In the binding assay, heparin beads were mixed with rCpTSP4 or GST and washed five times. The original solution (input), selected flowthroughs (Wash 2 and Wash 5), and the beads after washes (output) were fractionated in SDS-PAGE and analyzed by western blotting. In the competition assay, heparin-beads were incubated with rCpTSP4 in the presence of heparin or CSA at specified concentrations, washed, and analyzed by western blotting. (**G**) Binding kinetics of rCpTSP4 to heparin by enzyme-linked assay (App. *K*_d_ = 483 nM), in which heparin was coated in microplates for binding by rCpTSP4 and detection by ELISA-like procedures. This assay used an equimolar GST-tag as a negative control and for background signal subtraction. In **C **to **G**, GST-fused rCpTSP4 and GST-tag were detected using an anti-GST mAb and secondary antibodies conjugated with Alex Fluor-488 (**C and D**) or alkaline phosphatase (**E **to **G**). Error bars in **E **and **G **show the standard error of the mean (SEM; *N* = 3).

The binding property of CpTSP4 on host cells was investigated to gain insight into its biological roles using glutathione-S-transferase (GST)-fused recombinant CpTSP4 (rCpTSP4; excluding signal peptide) (Fig. 5A and B). rCpTSP4 could specifically bind to formalin-fixed HCT-8 cell monolayers as determined by flow cytometry and IFA (vs no binding by GST-tag) (Fig. 5C and D). The binding followed one-site-specific binding kinetics, showing nanomolar binding affinity (App. *K*_d_ = 334 nM) (Fig. 5E).

CpTSP4 contains four regions rich in basic amino acids (BAAs) (Fig. 5A), which is a common feature for HBMs (31, 32). We hence hypothesized that heparin/heparan sulfate (HS) might serve as the ligand (or one of the ligands) for CpTSP4. To test the hypothesis, we first performed a competition assay, showing that heparin, but not the closely related glycosaminoglycan chondroitin sulfate A (CSA), could compete with rCpTSP4 for the host cell-binding activity (Fig. 5E, inset). Second, direct binding of rCpTSP4 to heparin-conjugated agarose beads was observed in a column-based assay (vs no binding by GTS-tag) (Fig. 5F; upper panel). The binding of rCpTSP4 to heparin-beads could be specifically competed out by heparin but not by CSA (Fig. 5F; lower panel). Third, using an enzyme-linked microplate assay developed in this study, in which the microplates were coated with heparin (10 µM) and the binding of rCpTSP4 (vs GST-tag) was detected using anti-GST mAb, we observed one-site-specific binding kinetics of GST-CpTSP4 to heparin with sub-micromolar binding affinity (App. *K*_d_ = 483 nM) (Fig. 5G).

**Fig 6:**
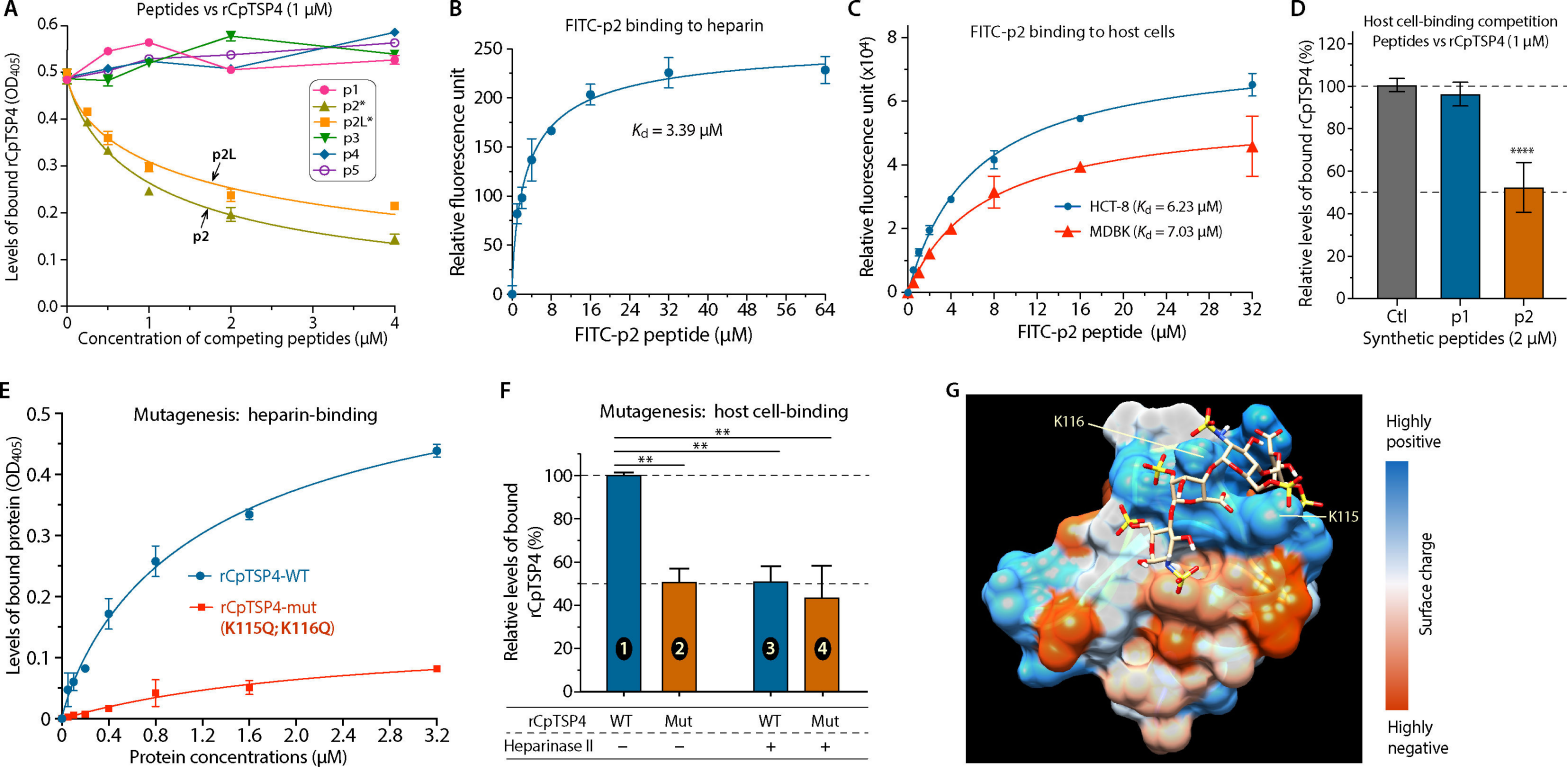
Determination of a novel HBM in CpTSP4 by peptide competition assay and site-directed mutagenesis. (**A**) Competition assay showing that the binding of rCpTSP4 to heparin coated in microplates could be competed out by p2 and p2L peptides in a dose-dependent manner but not by the other four synthetic peptides. Bound rCpTSP4 was detected by an anti-GST mAb similar to ELISA. (**B**) Binding kinetics of FITC-conjugated p2 peptide to heparin coated in microplates (App. *K*_d_ = 3.39 µM). (**C**) Binding kinetics of FITC-conjugated p2-peptide to formalin-fixed HCT-8 and MDBK cells (App. *K*_d_ = 6.23 and 7.03 µM, respectively). (**D**) The binding of rCpTSP4 (1.0 µM) to formalin-fixed HCT-8 cells was reduced by p2-peptide (2.0 µM) by 47.9% (vs 4.2% by the control p1-peptide). Ctl = control containing rCpTSP4 only; ****P < 0.0001 by the Sidak multiple comparison test. (**E**) Comparison of the heparin-binding kinetics between wild-type and mutated rCpTSP4 (rCpTSP4-WT vs rCpTSP4-mut), showing that the mutation of two Lys residues at the p2 site (K115Q/K116Q) eliminated the specific binding of rCpTSP4 to heparin in the enzyme-linked microplate assay. (**F**) The mutations (K115Q/K116Q) reduced the binding of WT rCpTSP4 to HCT-8 cells by 49.3% (bars 1 vs 2), similar to the 49.1% reduction caused by heparinase II treatment of host cells (bars 1 vs 3). Mutations at the p2-site (bars 2 and 4) or heparinase II treatment of host cells (bars 3 and 4) produced a similar level of reduction in the binding of rCpTSP4. **P < 0.01 by the Sidak multiple comparison test. (**G**) Molecular docking showing the binding of a tetrameric heparin to a positively charged crevice at the p2 site of CpTSP4, in which the Lys residues subjected to mutagenesis were indicated. Error bars = standard error of the mean (SEM; *N* = 3).

We then proceeded to identify the HBM in CpTSP4. Six peptides corresponding to the four BAA-rich sites in CpTSP4 were synthesized (see sequences and positions in Fig. 5A) and used in competition and binding assays. Site #2 was located in a PAN/APPLE domain and contained a relatively long BAA-rich sequence. Therefore, three peptides were synthesized, including a long peptide (p2L) and two short ones (p2 and p3). In a microplate-based competition assay, p2 and p2L peptides reduced the binding of rCpTSP4 to heparin in a dose-dependent manner (Fig. 6A), indicating that the p2 sequence was the HBM. Direct binding of p2-peptide to heparin was also confirmed, in which fluorescein isothiocyanate (FITC)-conjugated p2-peptide displayed low-micromolar binding kinetics to heparin in a microplate-based fluorescence assay (App. *K*_d_ = 3.39 µM) (Fig. 6B). FITC-p2 also displayed low-micromolar binding kinetics to host cells (App. *K*_d_ = 6.23 and 7.03 µM to HCT-8 and MDBK cells, respectively) (Fig. 6C). Additionally, p2-peptide (2 µM) could compete with rCpTSP4 (1 µM) for binding to HCT-8 cells (vs no competition by p1-peptide used as a control) (Fig. 6D).

To further validate that the p2 site sequence (^110^FFIKCKKWSYDVEKKK^125^) was responsible for the heparin binding of CpTSP4, site-directed mutagenesis was performed to substitute two Lys residues (containing a polar basic side chain) at positions 115 and 116 with two Gln residues (containing a polar neutral side chain) (i.e., K115Q and K116Q) (see illustration in Fig. 5A). In a microplate assay, the mutation essentially abolished the binding ability of rCpTSP4 to heparin (Fig. 6E). However, the mutated rCpTSP4 showed a low level of non-specific binding to heparin (Fig. 6E, red line), which was likely attributed to the electrostatic interactions between the clustered basic residues in rCpTSP4 and negatively charged heparin. In the host cell-binding assay, the mutations reduced the binding of rCpTSP4 to HCT-8 cells by ~50% (Fig. 6F, bars 1 vs 2). Pre-digestion with heparinase II to remove heparin from the surface of host cells also reduced the binding of rCpTSP4 to the host cells by ~50%. However, pre-digestion had no significant effect on the binding of the mutated protein to the host cells (that was already ~50% lower than the wild-type rCpTSP4) (Fig. 6F, bars 3 vs 4). We noticed that, under the physiological salt concentration (150 mM NaCl), non-specific binding of mutated rCpTSP4 to heparin or host cells was relatively high. This technical problem was solved using high-salt buffer (phosphate-buffered saline (PBS) containing 500 mM NaCl and 1% Tween-20; see more details in the Materials and Methods). High-salt buffer was used in all binding assays in this study to minimize the non-specific binding of rCpTSP4 to heparin or host cells. Heparin binding at the p2 site was also supported by molecular docking with a structural model comprised of 51 amino acids spanning the p2 site (amino acid positions 50 to 150) built by AlphaFold (33). The prediction of heparin binding used the ClusPro server (https://cluspro.bu.edu/) under the preset “heparin-binding mode” (34), in which the heparin tetramer could fit well into a positively charged crevice at the p2 site (Fig. 6G).

The discovery that CpTSP4 possesses heparin-binding properties expands the concept of heparin-binding proteins to include a protozoan TSP protein. The amino acid profile at the p2 site (XXXBxBBXxxnXnBBB; see annotation in Fig. 5A) also represents a novel HBM because it differs from known HBM consensus sequences (e.g., XBBXBX, XBBBXXBX, XBBBXXBBBXXBBX, or TXXBXXTBXXXTBB; B = basic, X = hydropathic, T = turn) (31, 32). Heparin and HS are present in the intestinal mucus and extracellular matrix (ECM) and are commonly explored as host ligands by microorganisms, including protozoan parasites, for attachment (35–37). Based on the peptide competition assay and binding kinetics of wild-type and mutated CpTSP4 (Fig. 6), the p2 site HBM might contribute roughly 50% of the binding activity of CpTSP4 to HCT-8 cells. Therefore, CpTSP4 would contain additional motif(s) for binding to other ligand(s) on host cells that remain to be determined.

## DISCUSSION

The early-branching *Cryptosporidium* is highly divergent from other apicomplexans in lifestyle (e.g., residing on top of host cells as an intracellular but extracytoplasmic parasite rather than inside host cells) and metabolism (e.g., lacking an apicoplast, a typical mitochondrion, and cytochrome-based respiratory chain) (38, 39). *Cryptosporidium* also possesses two unique central microtubules with unknown biological roles but lacks the SPMT network (18). This study suggests that the two *C. parvum* microtubules are involved in the anterograde trafficking of a micronemal protein. The two microtubules appear to be attached to γ-tubulin-based MTOC, which differs from SPMT in *Toxoplasma* and *Plasmodium*, which use an apical polar ring as “unconventional” MTOC to form a network to confer the elongated shape of the zoites (25, 40). The two central microtubules are unseen in other apicomplexan lineages (e.g., coccidia and hematozoa). Therefore, their involvement in intracellular trafficking of a micronemal protein is not a common feature shared by apicomplexans.

Besides CpTSP4, only a limited number of other micronemal proteins have been experimentally confirmed by IFA and/or IEM for anterior locations in sporozoites. These include CpGP900 (cgd7_4020), TRAPC1 (cgd1_3500), CpTSP8 (cgd6_780), CpROM1 (cgd3_980), cgd1_3550 (APPLE domain-containing protein), cgd2_1590 (APPLE/EGF-like domain-containing protein), and cgd7_1960 (WD40/YVTN repeat-like domain-containing protein) (9–11, 23). Among them, cgd7_1960 was more recently discovered as one of the proteins in the “microneme cluster” by HyperLOPIT technology and confirmed by IFA of hemagglutinin (HA)-tagged transgenic *C. parvum* (11). This HyperLOPIT-based study identifies several new candidate micronemal proteins based on their clustering with known microneme markers (e.g., CpGP900). It also clarifies that the previously suggested micronemal protein Cpa135 (cgd7_1730) is actually localized to the crystalloid body at the sporozoite posterior end. Nonetheless, these micronemal proteins show no filamentous distributions in sporozoites, suggesting that not all micronemal proteins utilize the two central microtubules for trafficking. This seems reasonable, as many micronemal proteins are apically discharged.

The requirement of microtubules in the secretion of CpTSP4 is supported by IFA (distribution on the two microtubules) and by inhibition assay (complete inhibition of secretion by SB-743921 and SB-715992, the two selective kinesin-5/Eg5 inhibitors known for no off-target effect). Current data suggest anterograde transport of CpTSP4 in sporozoites, implying that CpTSP4 may be secreted from the posterior end and/or peripherally. However, more direct evidence is needed to firmly determine where and how CpTSP4 is discharged from the zoites. We are also unable to exclude the possibility that CpTSP4 may be apically secreted, using other types of microtubules that are unrecognizable by our anti-CpTubB antibody. Indeed, several short microtubule-like structures are observable on the apex of *C. parvum* sporozoites (41). The biological roles and potential involvement of these apical microtubules in the secretion of CpTSP4 remain to be elucidated. For comparison, *Toxoplasma* and *Plasmodium* zoites contain short intraconoidal microtubules that are implied for involvement in the transport of vesicles (6, 8).

Our data imply that CpKin5 is the possible molecular motor involved in the trafficking of CpTSP4-cargoes on the two central microtubules. However, a more thorough investigation is required to pin down the molecular trafficking mechanism of protein cargo transport in *Cryptosporidium*, including the anterograde transport of CpTSP4-cargoes towards the posterior end of the two central microtubules. It is worth generating new antibodies against CpKin proteins, including CpKin5, for immuno-colocalization with CpTSP4 and microtubules. It is also necessary to test whether only CpKin5, among all CpKin proteins, is sensitive to SB-743921 and SB-715992. While SB-743921 and SB-715992 could completely block the secretion of CpTSP4, another human kinesin-5/Eg5 inhibitor, monastrol, is ineffective, implying that CpKin5 might share insufficient tertiary structural similarity with human kinesin-5/Eg5 at the monastrol-binding pocket.

This study revealed a novel HBM responsible for part of the adhesive property of CpTSP4 to host cells. The other adhesive domain(s) in CpTSP4 and corresponding host ligand(s) remain to be determined. CpTSP4 lacks TMDs and is absent on the zoite plasma membranes, as shown by IFA. Physiochemically, CpTSP4 is neutrally charged (pI = 7.45), thus unable to adhere to sporozoites or host cells by simple electrostatic interaction. Therefore, the released CpTSP4 would mostly adhere to the heparin- and HS-containing mucus and ECM, making no or little contribution to the adherence of the zoites. We speculate that the secreted CpTSP4 adhering to the host cell mucus/ECM may serve as a molecular cushion to minimize direct contact of moving zoites with the intestinal mucins. This is beneficial to the moving zoites because they avoid being restrained by the negatively charged mucins.

Like CpGP900, CpTSP4 is absent in the sexual stages, suggesting that the two micronemal proteins play no role in the movement of male gametes. In fact, both female and male gametes lack microneme-like structures (42, 43), suggesting that male gametes may not use micronemes to store or discharge proteins. During their motility to reach the immotile female gametes, male gametes are expected to secrete adhesive and lubricative molecules. Therefore, there would be other secretory pathways in the gametes that are independent of the micronemal secretion.

As globally distributed enteric parasites of medical and veterinary importance, only a single drug (nitazoxanide) is Food and Drug Administration-approved for treating cryptosporidiosis in immunocompetent patients. There is a lack of effective drugs for use with immunocompromised patients (3, 44). Kinesin-5/Eg5 has been an effective target for developing anticancer therapeutics and has been explored as an anti-malarial target (45). This study shows that kinesin-5/Eg5 inhibitors could fully block the secretion of CpTSP4. Whether the two inhibitors and derivatives could be explored or repurposed for developing anti-cryptosporidial drugs requires further investigation. However, CpKin5 shows a sufficient sequence difference from human kinesin-5/Eg5 (73.3%/55.2% similarity/identity), suggesting that highly selective anti-CpKin5 inhibitors could be potentially developed for anti-cryptosporidial therapeutics or for studying the biology of the kinesin-5 ortholog in the parasite.

## MATERIALS AND METHODS

### Parasites and cell lines

A *C. parvum* isolate with the gp60 subtype IIaA17G2R1 propagated in calves was used in all experiments. Oocysts were purified by sucrose gradient centrifugation protocols (46, 47) and stored in PBS containing 200 units/mL penicillin and 0.2 mg/mL streptomycin at 4°C. A human ileocecal epithelial cell line (HCT-8; ATCC # CCL-244) and a bovine kidney cell (MDBK; ATCC # CCL-22) were cultured in RPMI-1640 medium containing 10% fetal bovine serum (FBS), 2 mM L-glutamine, 50 units/mL penicillin, and 50 µg/mL streptomycin at 37°C in 5% CO_2_. For binding or infection assays, host cells were seeded into 24-, 48-, or 96-well plates and allowed to grow overnight (~80% confluence). For IFA, host cells were cultured in 48-well plates containing poly-L-lysine-treated round glass coverslips. The parasite invasion stage was prepared by infecting HCT-8 cell monolayers (~60% confluence) with freshly excysted sporozoites for 15 to 30 min (9, 18, 23). Intracellular stages were prepared by inoculating HCT-8 monolayers with bleached and washed oocysts (*in vitro* excystation rate >80%) at 37°C for 3 h, followed by the removal of uninvaded parasites by medium exchange and continuous cultivation for various times as specified.

### Antibody preparation

Anti-CpTSP4 mouse mAb was produced against a unique peptide (^89^KIKKADSWQEC^99^). Two specific pathogen-free mice were immunized five times with keyhole limpet hemocyanin-linked peptide, including 100 µg with Freund’s complete adjuvant for the first intradermal injection, 50 µg with incomplete adjuvant for next three intradermal injections, and 20 µg without adjuvant in the final intravenous injection. Standard protocols were used in hybridoma production (48, 49). After three rounds of ELISA screening, the clone 9E6 (subtype IgG1) was used for producing mouse ascites (anti-CpTSP4 mAb/clone 9E6). Non-immunoglobins in the ascites were removed by precipitation with caprylic acid, and immunoglobins were concentrated by ammonium sulfate precipitation (50). Purified mAb was stored at 2 µg/µL. Rabbit anti-CpGP900-C pAb and anti-CpTubB pAb were prepared and affinity-purified as described (18, 23).

### Western blot analysis

*C. parvum* sporozoites were lysed in radioimmunoprecipitation assay buffer containing a protease inhibitor cocktail for mammals (Sigma-Aldrich). Sporozoite lysates (~7.0 × 10^7^/lane) in reducing sample buffer were heated at 95°C for 5 min, followed by fractionation in SDS-PAGE and transfer to nitrocellulose membranes. The blots were blocked in 5% bovine serum albumin (BSA) in a buffer containing 50 mM Tris-HCl at pH 7.5, 150 mM NaCl, and 0.05% Tween-20 (TBST buffer) for 1 h and probed in 5% BSA-TBST buffer containing anti-CpTSP4 mAb (1:50 dilution) for 1 h. After three washes with TBST, blots were incubated with horseradish peroxidase-conjugated goat anti-mouse IgG (Invitrogen, Waltham, MA, USA) and visualized using an enhanced chemiluminescence reagent (Beyotime Biotechnology, Shanghai, China). All procedures were conducted at room temperature unless specified.

### Indirect immunofluorescence assay

Intact oocysts suspended in 4% paraformaldehyde were ruptured by three freeze-and-thaw cycles between liquid nitrogen and ice, followed by incubation for 30 min on ice and three washes with PBS. Excysted sporozoites were prepared by incubating oocysts in excystation medium (RPMI-1640 medium containing 0.75% taurodeoxycholic acid). Free merozoites were isolated from the supernatants of HCT-8 monolayers infected with *C. parvum* for 18–20 h. Intracellular parasites cultured on coverslips for various times were prepared as described above. Sporozoites and merozoites in suspension were fixed with cold 4% paraformaldehyde for 30 min and washed with PBS. Ruptured oocysts, sporozoites, and merozoites after fixation were applied to poly-L-lysine-coated glass slides. Samples were air-dried for 1 h, permeabilized with 0.2% Triton X-100 for 5 min, and blocked for 1 h in 5% milk-PBS prepared from non-fat dry milk. Intracellular parasites on coverslips were similarly fixed, permeabilized, and blocked. In gliding trail experiments, sporozoites after excystation were resuspended in RPMI-1640 medium, placed on poly-L-lysine-coated slides, and incubated for 20 min at 37°C, followed by the same fixation, permeabilization, and blocking procedures.

After blocking, specimens were incubated with specified antibodies in PBS containing 500 mM NaCl (high-salt PBS to minimize non-specific binding) for 1 h, washed three times with PBS, and incubated for 1 h with appropriate secondary antibodies conjugated with specified fluorophores (Thermo Scientific, West Palm Beach, FL, USA), including the Alexa Fluor 488-labeled donkey anti-mouse-IgG antibody and the Alexa Fluor 594-labeled goat anti-rabbit-IgG antibody. In some specimens, *Vicia villosa* lectin conjugated with Texas red was used to outline the parasite gamonts. All secondary antibodies were pre-tested to confirm that they were not cross-reactive with *Cryptosporidium* specimens. For invasion stage specimens, host cell F-actin was co-stained using rhodamine-phalloidin (Solarbio, Beijing, China). Samples were counter-stained for nuclei with 4,6-diamidino-2-phenylindole (Sigma-Aldrich; 1.0 µg/mL). Procedures were performed at room temperature. Slides were examined under an Olympus BX53 research fluorescence microscope. Images were captured with an Olympus DP72 camera and stored in TIFF format. The relative fluorescence intensities were measured in some sporozoite samples and analyzed using ImageJ (Fiji). Images were processed with Adobe Photoshop (v2021 or higher), in which the signal levels might be linearly adjusted without local manipulations.

### ELISA detection of CpTSP4 secreted from sporozoites

The relative levels of CpTSP4 secreted from and retained in sporozoites during and after the excystation were detected by ELISA. Excystation was performed by incubating *C. parvum* oocysts (3 × 10^6^) in 75 µL excystation medium at 37°C for 45 min or as specified. In some assays, selected dynein and kinesin inhibitors at specified concentrations were added into the excystation medium containing 0.5% DMSO to evaluate their effects on the secretion of CpTSP4. Samples were centrifuged at 12,000 ×*g* for 5 min to collect supernatants and pellets. After excystation and medium removal, some sporozoites were further incubated in 75 µL RPMI-1640 medium at 37°C for 60 min to collect supernatants and pellets. Sporozoite pellets were resuspended in 75 µL RPMI-1640 medium and subjected to five freeze-and-thaw cycles between liquid nitrogen and ice, followed by centrifugation for collecting lysates. For comparison, intact oocysts (3 × 10^6^) in 75 µL RPMI-1640 medium were subjected to the same freeze-and-thaw cycles and centrifugation to collect supernatants. These supernatants, including blank controls (plain medium or medium containing 0.5% DMSO), were mixed with an equal volume of coating buffer (0.05 M carbonate-bicarbonate, pH 9.6). All samples were calibrated to contain 0.375% sodium taurocholate.

Samples in coating buffer were added into 96-well plates in triplicate (50 µL/well, each equivalent to 10^6^ oocysts) and incubated overnight at 4°C. Plates were rinsed with 0.05% Tween-20/PBS and blocked with 5% milk/PBS (100 µL/well) at 37°C for 1 h. After three washes with 0.05% Tween-20/PBS, plates were incubated with anti-CpTSP4 mAb (2 µg/µL; 1:40 dilution) in high-salt PBS containing 500 mM NaCl (50 µL/well) at 37°C for 1 h. For reference and as a positive control, a rabbit antiserum (1:40 dilution) against *C. parvum* total sporozoite proteins was used in parallel for all samples. After three washes with high-salt wash buffer, plates were incubated with alkaline phosphatase-conjugated goat anti-mouse IgG or anti-rabbit IgG (H + L) secondary antibodies (ImmunoWay Biotechnology, Plano, TX, USA; 1:10,000 dilution), followed by color development with p-nitrophenyl phosphate (Sigma-Aldrich). Optical density at 405 nm (OD_405_) was measured in a multifunctional microplate reader (BioTek, VT, USA).

### Heterologous expression of recombinant CpTSP4 and site-directed mutagenesis

CpTSP4 without signal peptide (cgd8_150; amino acids 26 to 488) was expressed as GST-fusion protein (rCpTSP4). A gene fragment was amplified from *C. parvum* genomic DNA by high-fidelity PCR using primers CpTSP4-BamHӀ-F (5′-CGCGGATCCAAATACATTACCCCAGAAAAA-3′) and CpTSP4-EcoRӀ-R (5′-CCGGAATTCTTAGTTGAACAAATTTGGATCTA-3′) (underlines indicate restriction enzyme sites). Amplicons were cloned into a pGEX-4T-1 expression vector (Invitrogen) at specified restriction sites. GST-tag alone was expressed using a blank pGEX-4T-1 vector and used as a negative control. In the HBM validation assay, two Lys residues were replaced by Gln (K115Q/K116Q) by site-directed mutagenesis using the following primers in overlapping PCR: (1) CpTSP4-mut-BamHӀ-F1 (5′-GTGGATCCAAATACATTACCC**C**AG**A**AAAA-3′) and CpTSP4-mut-R1 (5′-ATAGCTCCATT**G**CT**G**ACACTTAATAAAGAAGAATGCA-3′); (ii) CpTSP4-mut-F2 (5′-CTTTATTAAGTGT**C**AG**C**AATGGAGCTATGATGTAGAA-3′) and CpTSP4-EcoRӀ-R2 (5′-CCGGGAATTCTTAGTTGAACAAATTTGGATCTA-3′) (bold fonts indicate mutation sites; underlines indicate restriction sites). All recombinant proteins were expressed in *Escherichia coli* strain BL21(DE3) cells and purified using a glutathione-sepharose 4B column following standard protocols. The purity and molecular weight were evaluated with SDS-PAGE.

### Host cell-binding assays

The host cell binding of rCpTSP4 (wild-type or mutated) was evaluated by immunofluorescence-based, flow cytometric, and enzyme-linked assays. An equimolar GST-tag was used as a negative control. In an immunofluorescence-based binding assay, HCT-8 cells were cultured to full confluence on coverslips placed in 48-well plates, fixed with 4% paraformaldehyde in PBS for 30 min on ice, blocked with 5% milk-PBS for 1 h, incubated with wild-type or mutated rCpTSP4 or GST-tag (each at 1.0 µM) in high-salt binding buffer (PBS containing 1.0 mM CaCl_2_, 1.0 mM MnCl_2_, and 500 mM NaCl) overnight at 4°C, fixed again with cold methanol for 10 min, and blocked again with 5% milk-PBS for 1 h. There were three washes of PBS after each step. Proteins bound to the host cells were detected using anti-GST mAb (ImmunoWay Biotechnology) at 37°C for 1 h, followed by incubation with Alexa Fluor 488-labeled donkey anti-mouse-IgG antibody. Samples were examined under a fluorescence microscope as for the IFA described above.

A flow cytometric assay was performed as described (51). Briefly, HCT-8 cells were cultured to semi-confluence at 37°C, washed twice with PBS, and treated with 500 µM EDTA in PBS for 15 min at 37°C. Detached cells (~2 × 10^6^ in 400 µL) were washed with 2% FBS-PBS and incubated sequentially with 7.5 µM rCpTSP4 or GST-tag in 2% FBS-PBS at 4°C for 2 h, anti-GST mAb at 4°C for 1 h, and Alexa Fluor 488-labeled Donkey anti-mouse-IgG antibody at 1:1,000 dilution for 4°C for 1 h. There were three washes with PBS after each step. At least 10,000 cells were analyzed with the FACSCalibur system (BD Bioscience, Franklin Lakes, NJ, USA).

Enzyme-linked assays used previously described ELISA-like procedures (52–54). Briefly, HCT-8 cells were cultured to confluence in 96-well plates, fixed with 1% glutaraldehyde in PBS for 30 min, blocked with 5% milk-PBS for 1 h at 37°C, and treated with rCpTSP4 or GST-tag in 500 mM NaCl-PBS at specified concentrations overnight at 4°C. In some experiments, 20 µM heparin (substrate competitor; Sigma-Aldrich, # H9399-5MU) or 20 µM CSA (negative control; Sigma-Aldrich, # C9819-5G) was mixed with 2.0 µM rCpTSP4 (or GST-tag) in 500 mM NaCl/PBS and then added to the fixed HCT-8 cells for 1 h at 37°C. Some HCT-8 cell specimens were treated with heparinase II (1.0 U/mL) (Sigma-Aldrich) in RPMI-1640 for 1 h at 37°C, followed by fixation with 1% glutaraldehyde for 30 min, three washes in PBS, and incubation with 1.0 µM rCpTSP4 or GST-tag. Proteins on host cells were detected using ELISA procedures as described above.

### Microplate heparin-binding assays

Heparin binding of CpTSP4 was evaluated by microplate-based and column/resin-based binding assays. The microplate assay resembles ELISA, in which 96-well plates were coated with heparin in 0.05 M carbonate-bicarbonate (pH 9.6) overnight at 4°C and blocked with 5% milk-PBS for 1 h at 37°C. In determining the binding kinetics, heparin-coated plates were incubated with serially diluted rCpTSP4 or rCpTSP4-mut (K115Q/K116Q), or GST-tag control, in high-salt PBS containing 500 mM NaCl and 1% Tween-20 (PBST) for 1 h at 37°C. In the peptide competition assay, plates were incubated with 1.0 µM rCpTSP4 or GST-tag premixed with serially diluted synthetic peptides (HBM candidates) for 1 h at 37°C. The subsequent procedure followed those for standard ELISA, as described above.

A column-based binding assay used heparin-agarose beads (GE Healthcare) as described (51, 55). Briefly, GST-CpTSP4 or GST-tag (1.0 µM or as specified) with or without heparin or CSA (1.0 µM) in PBS was incubated with 40 µL heparin-agarose beads at 4°C for 2 h, washed five times with PBS, and boiled for 5 min in loading buffer. After quick centrifugation, supernatants were fractionated by SDS-PAGE, transferred onto a nitrocellulose membrane, and probed with an anti-GST antibody using standard western blot procedures.

### Evaluation of the binding kinetics of the p2-peptide to host cells and heparin

Heparin-coated microplates and formalin-fixed HCT-8 monolayers were prepared as described above. These microplates were blocked in 5% milk/PBS and incubated with FITC-conjugated p2-peptide (FITC-p2) at specified concentrations under the same experimental conditions for assaying the binding of rCpTSP4. After washes with PBST buffer, relative fluorescence signals from FITC-p2 bound to heparin or host cells were detected using a BioTek multifunctional microplate reader (*λ*_EX_ = 490 nm; *λ*_EM_ = 520 nm).

### Molecular docking

For the binding of CpKin5 to SB-743921, the CpKin5 model was retrieved from the AlphaFold Protein Structure Database (A3FPW8; https://alphafold.ebi.ac.uk/). Using UCSF Chimera (56), the 3D structure of the conserved N-terminal motor domain (amino acids from 1 to 371) showed high confidence (per-residue confidence score pLDDT >90) and was aligned and superimposed with a model of the human Eg5-SB-743921 complex (PDB ID: 4BXN) (28). For heparin-CpTSP4 binding, a 51-aa region at the p2 site (^50^ASLCMDYVAFFFIKC**KK**WSYDVEKKKCLIKSGDRLCKYPD ENYISGLKNA S^101^; underline indicating HBM and bold fonts indicating critical residues) was extracted from the model (Q5CQ00). The heparin binding was predicted using the ClusPro 2.0 server under the parameters specified for heparin binding (34). Models were displayed using UCSF Chimera (56).

### Statistical analysis

Quantitative experiments were performed three or more times independently, with at least three biological replicates and two technical replicates. Charts and statistical analysis used GraphPad Prism (v9.0 or higher; San Diego, CA, USA). Statistical significance was evaluated by a two-tailed Student’s *t*-test for two-group data or one- or two-way ANOVA with specified multiple comparisons for multi-group data as described in the Results section and figure legends.

### Animal studies

Animal experiments were approved by the Animal Welfare and Research Ethics Committee of Jilin University (AUP # 2020-1Z-20). Specific pathogen-free (SPF) female BALB/c mice (6 to 8 weeks old) and female rabbits were used in producing monoclonal or polyclonal antibodies. All animals were housed in an institutionally approved facility.

## SUPPLEMENTAL MATERIAL

Figures S1 to S9 mbio.03158-23-s0001.pdf Additional immunostaining images, sequence alignments, and molecular docking models.

Table S1 mbio.03158-23-s0002.pdf List of TSP/TRAP family proteins in *Cryptosporidium parvum*.

Table S2 mbio.03158-23-s0003.pdf Similarity and identity scores of the motor domains between the seven *C. parvum* kinesin superfamily proteins.

Table S3 mbio.03158-23-s0004.pdf Similarity and identity scores between kinesin-5 motor domains from selected species.

ASM does not own the copyrights to Supplemental Material that may be linked to, or accessed through, an article. The authors have granted ASM a non-exclusive, world-wide license to publish the Supplemental Material files. Please contact the corresponding author directly for reuse.
