# Supplementary material for: Requirement of microtubules for secretion of a micronemal protein CpTSP4 in the invasive stage of the apicomplexan Cryptosporidium parvum
Source: mBio. 2024 Jan 24;15(2):e03158-23. doi: 10.1128/mbio.03158-23 (PMC10865969; doi:10.1128/mbio.03158-23)

Fig. S1.

Additional images on immunostaining of CpTSP4 in *Cryptosporidium parvum* oocysts ruptured by repeated freeze-and-thaw cycles. A. Immunostaining using an anti-CpTSP4 mAb alone (green); B. Dual-labeling with anti-CpTSP4 mAb (green) and rabbit anti-CpGP900 pAb (marker of micronemes; red). DIC, differential interference contrast microscopy; Nuc, nuclei counter-stained with DAPI (blue); mAb, monoclonal antibody; pAb, polyclonal antibody.

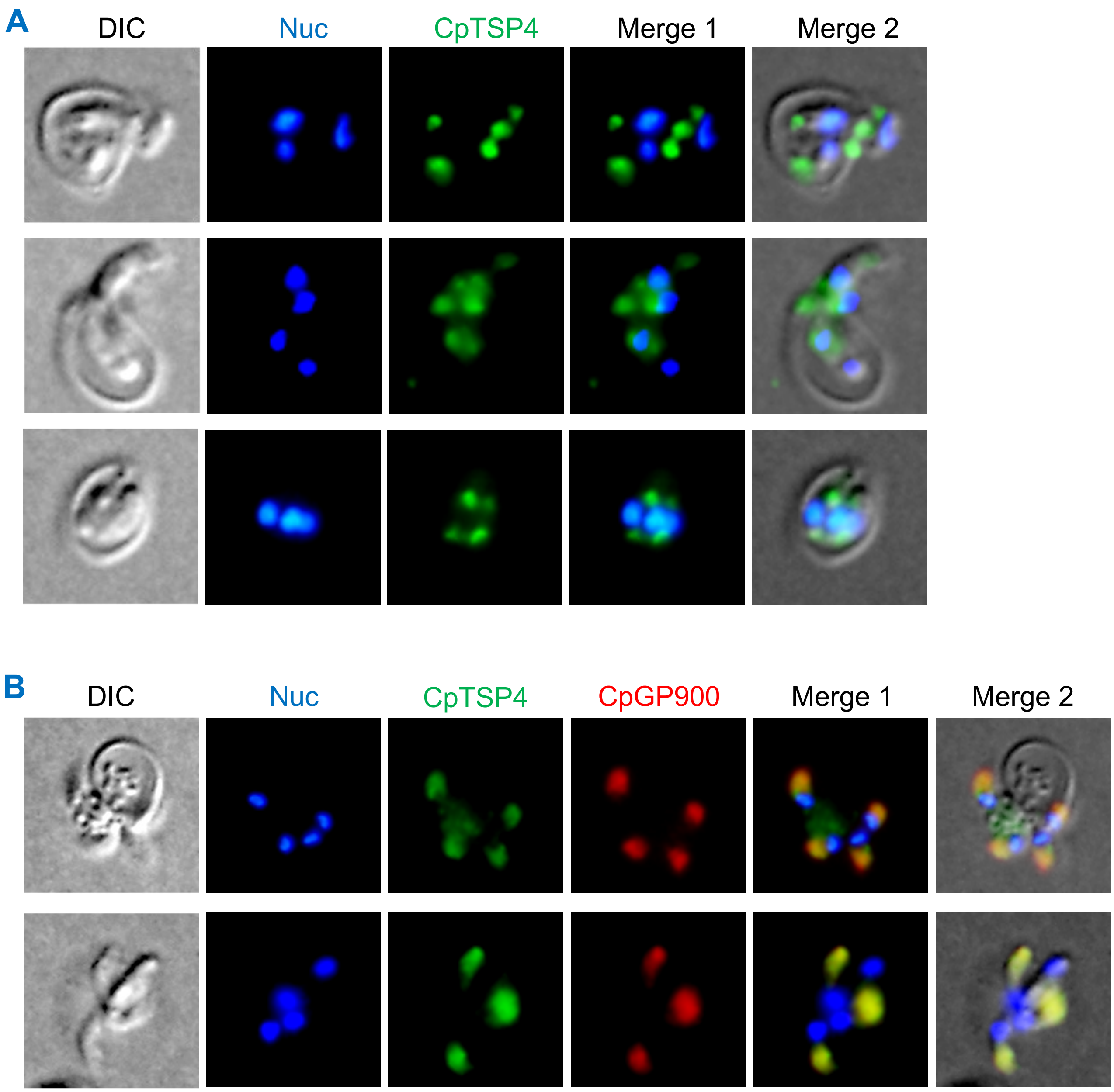

Fig. S2.

Additional images of dual-labeling IFA of CpTSP4 with CpTubB in *C. parvum* excysted sporozoites using anti-CpTSP4 mAb (green in **A** and red in **B**) and rabbit anti-CpGP900 pAb (red in **A** and green in **B**). DIC, differential interference contrast microscopy; Nuc, nuclei counter-stained with DAPI.

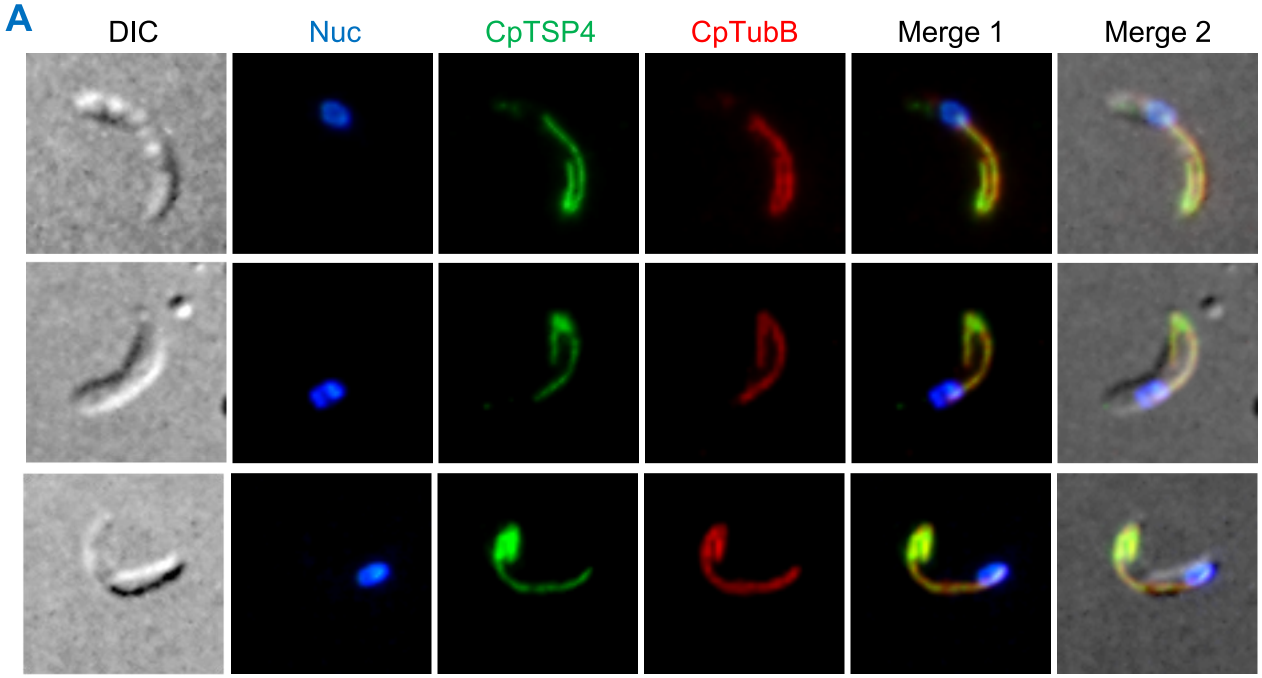

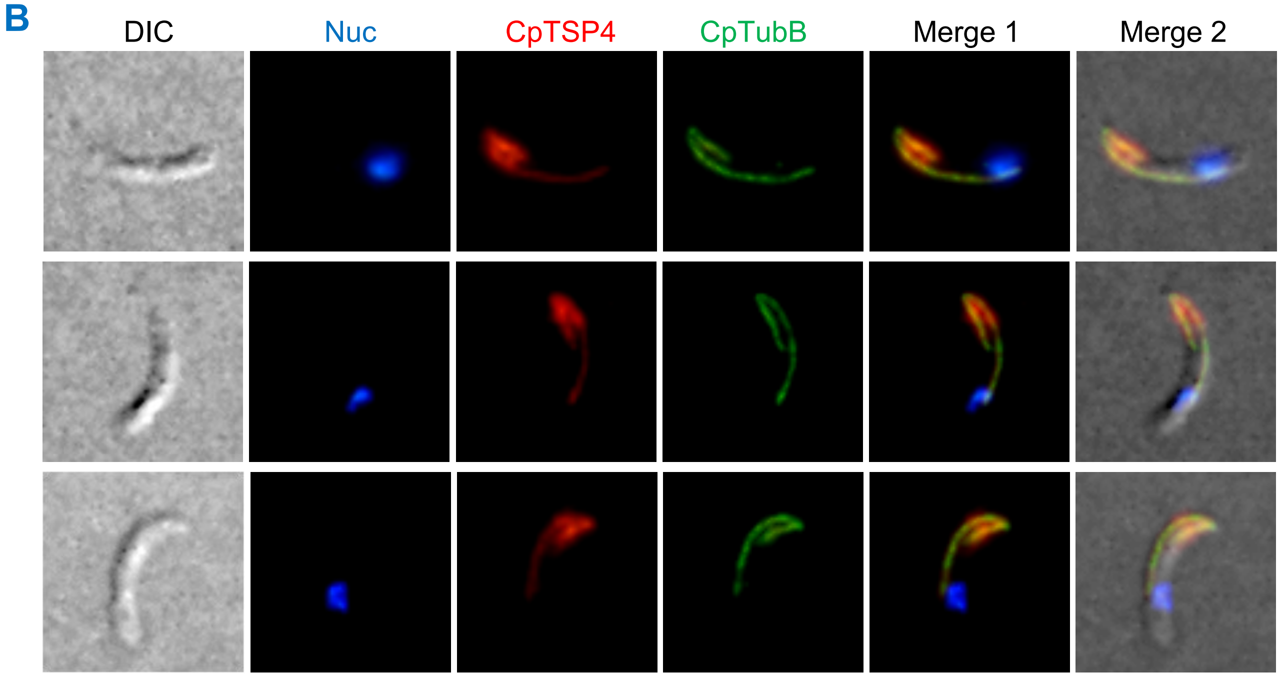

Fig. S3.

Validation of antibody specificity in IFA for potential cross-reactions between CpTSP4 and CpTubB. When anti-CpTubB pAb (upper panel) or anti-CpTSP4 mAb (lower panel) was individually pre-incubated with the two peptide immunogens, CpTubB peptide eliminated the fluorescent signals from anti-CpTubB pAb (upper panel), but not the signals from anti-CpTSP4 mAb (lower panel), whereas CpTSP4 peptide eliminated the signals from anti-CpTSP4 mAb (lower panel), but not the signals from anti-CpTubB pAb (upper panel).

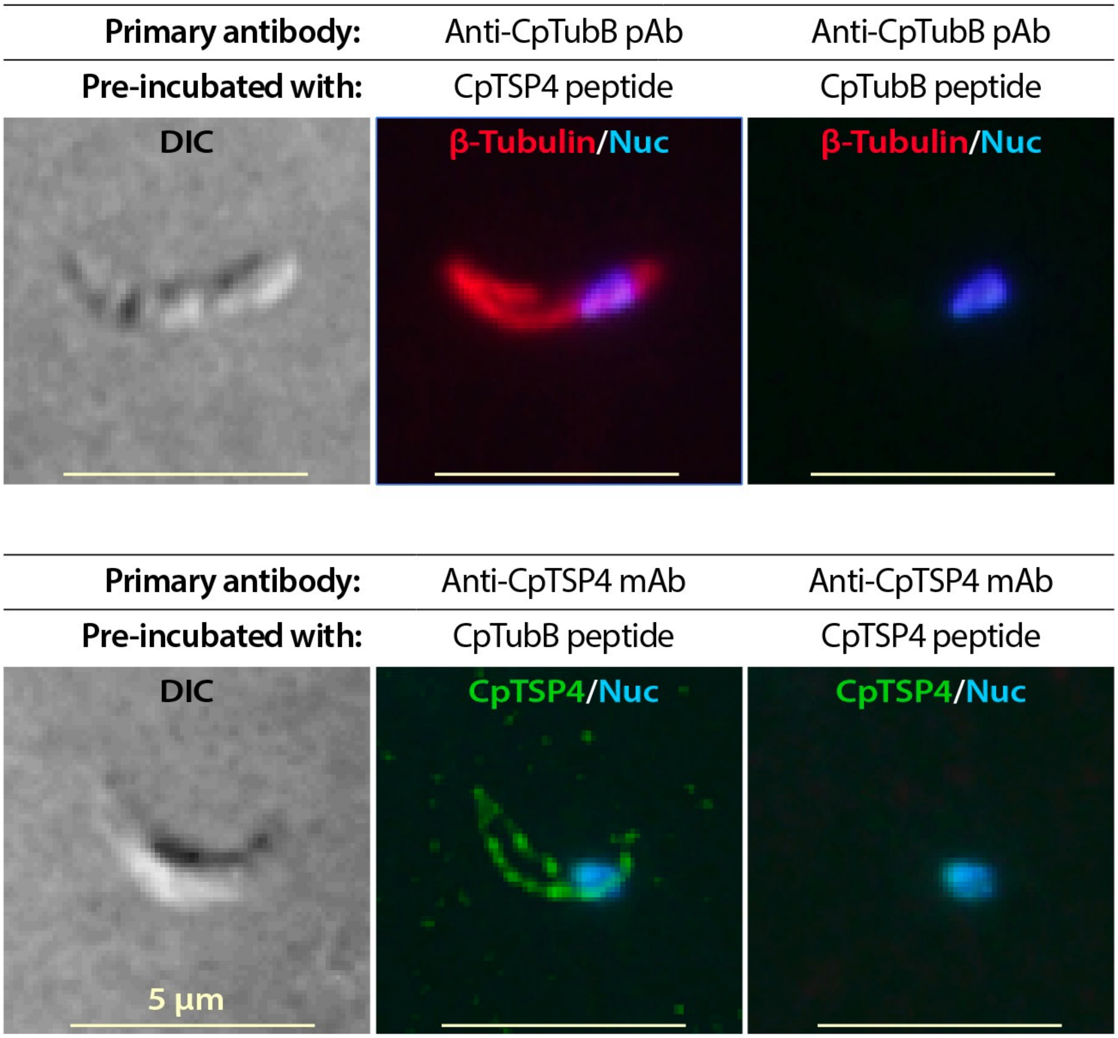

Fig. S4. Additional images on immunostaining of CpTSP4 (green) in gliding sporozoites using anti-CpTSP4 mAb alone (**A**) or colocalized with rabbit pAb against total sporozoite proteins (CpTPs; red) (**B**), showing the presence of granular CpTSP4 in the gliding trails. DIC, differential interference contrast microscopy; Nuc, nuclei counter-stained with DAPI.

A

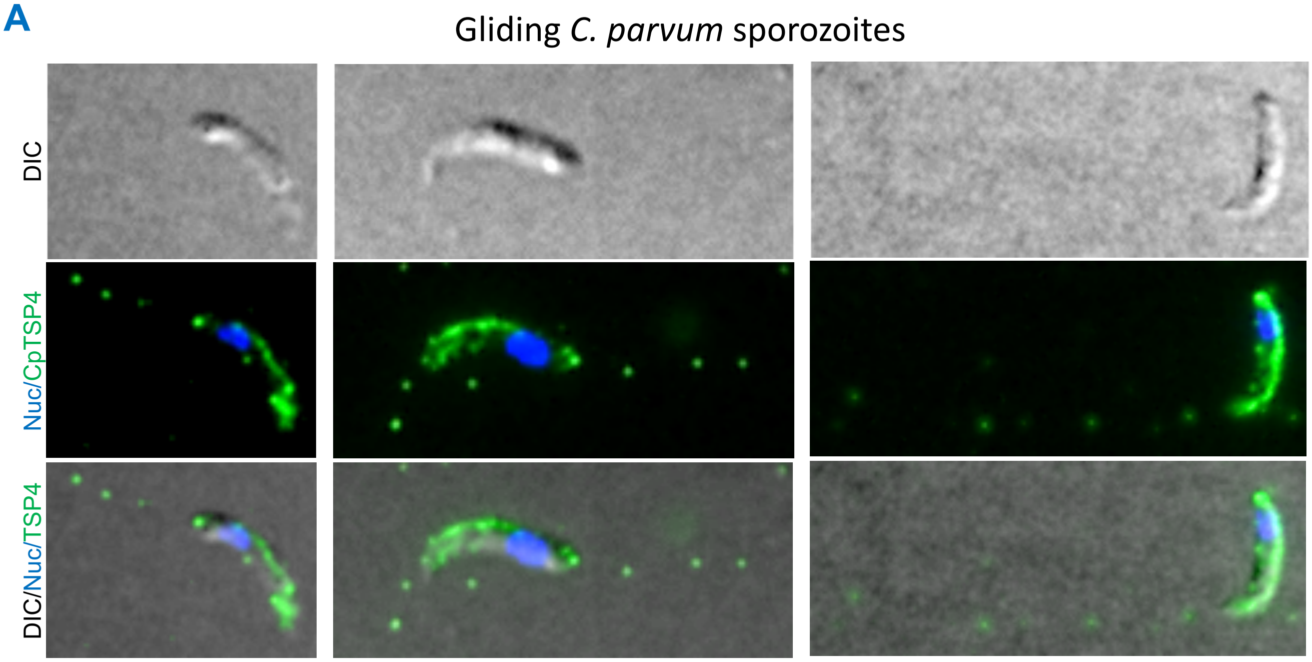

B

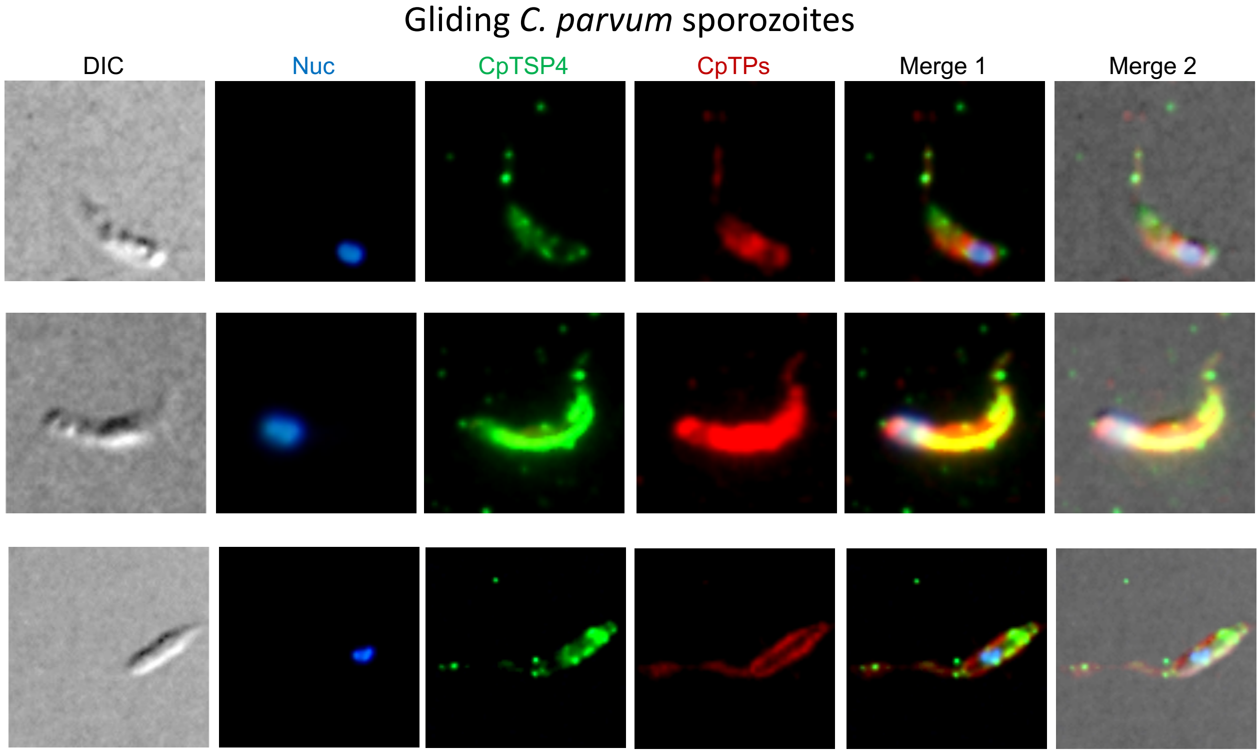

Fig. S5. Additional images on immunostaining of CpTSP4 (green) in intracellularly developing *C. parvum* using anti-CpTSP4 mAb. DIC, differential interference contrast microscopy; Nuc, nuclei counter-stained with DAPI.

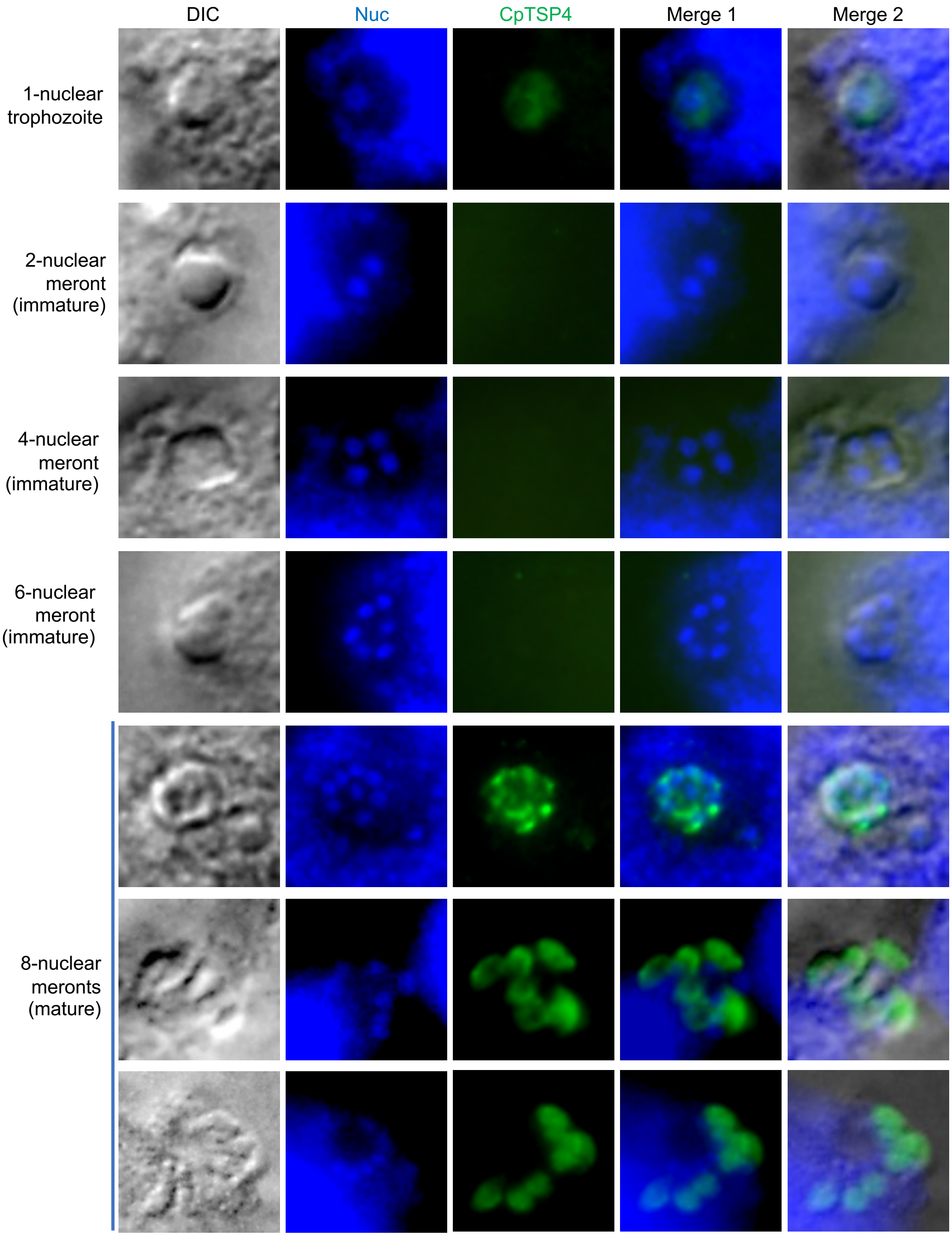

Note:

1) The 1-nuclear trophozoite was transformed from sporozoites shortly after invasion, thus containing some residual CpTSP4 displaying weak signals.
2) Immauture meronts are multi-nuclear cells (i.e., single cytoplasma containing 2 to 8 nuclei), in which banana-shaped merozoites had not formed yet. Therefore, they contained no micronemes.
3) The 8-nuclear meront (mature) contained well-developed merozoites that contain micronemes. Nuclei might be present at different focal points, for which not all nuclei were well shown in individual images.

Fig. S6. Additional images on immunostaining of CpTSP4 (green) in free merozoites isolated from the culture medium at 18 to 20 h post-infection time using anti-CpTSP4 mAb, showing the presence of CpTSP4 in micronemes and along the two central microtubules. DIC, differential interference contrast microscopy; Nuc, nuclei counter-stained with DAPI.

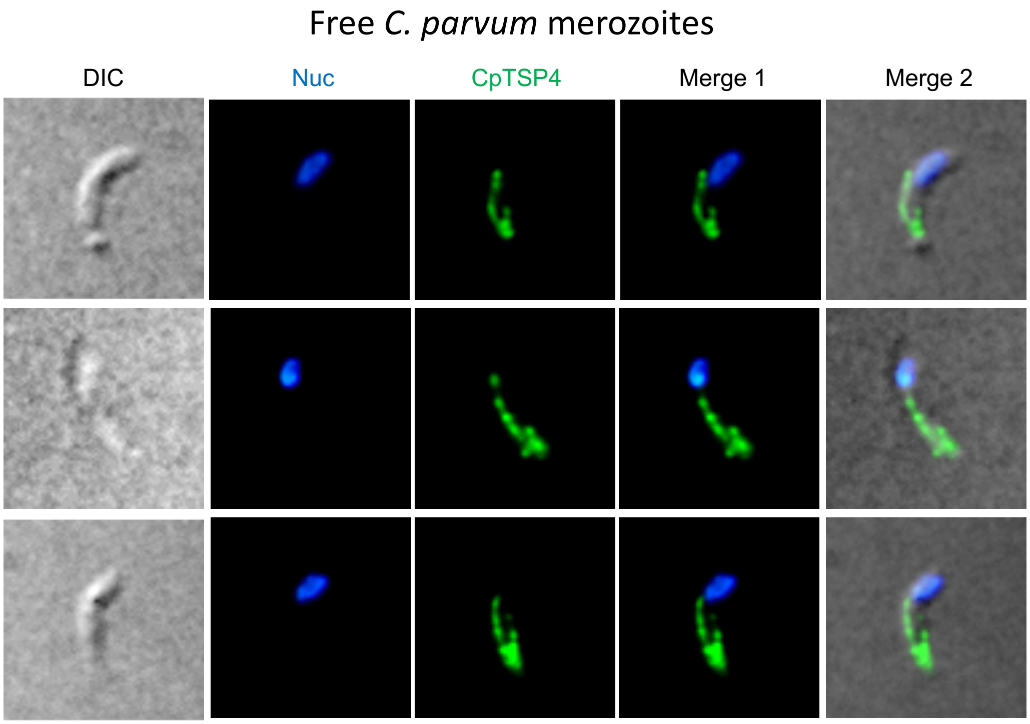

Fig. S7. Additional images on the effect of the selective kinesin-5 inhibitor (SB723921) on the distribution of CpTSP4 (green) in sporozoites during excystation. Oocysts were incubated in excystation medium in the absence or presence of inhibitors (50 μM) at 37 C for 1 h, followed by fixation and immunostaining with anti-CpTSP4 mAb. The treatment of the two inhibitors resulted in the accumulation of CpTSP4 in the sporozoite anterior region and disappearance of signals on the two microtubules. DIC, differential interference contrast microscopy; Nuc, nuclei counter-stained with DAPI.

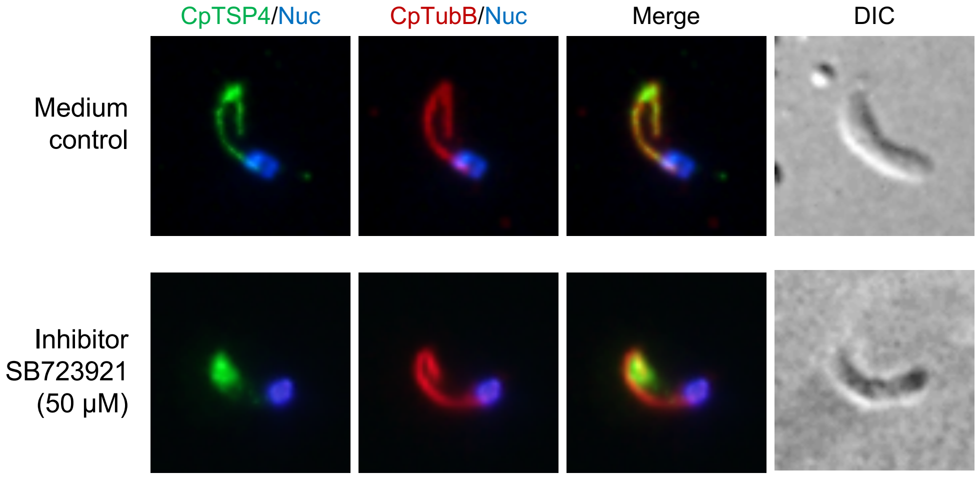

Fig. S8. Multiple sequence alignment of *C. parvum* kinesin-5 ortholog (CpKin5, cgd6_4210) with those from human, yeast and selected other apicomplexan species.

```
                               10 20 30 40 50 60 70 80 90 100
cgd6_4210_C_parvum             MKNSKPRQNTIEENGVNVKVIVRCRPLTEQEKKDPSNSNVLQVKPDSKEIVVSHQS------------------------LSRKFDSYSTKLFTFDGVCGSFTSQRE
CMU_020550_C_muris             MKPNRTKFNVSDENGVNVKVIVRCRPSTEQEKKDPSNFNILQTRPETKEIVVSQQG------------------------LGRKIDSYSSKVFSFDGVCGAYTSQKE
PF3D7_0317500_P_falciparum     --MLRNSYNNDKSSCVNIKVIVRCRPLNEKEKNDINNEEVVRINNNEVILTINRN------------------------------NEIYEKKYSFDYACDKDVDQKT
TGME49_297110_T_gondii         CLAPRKMPSAGGDCRTNVKVVVRCRPMTAVEQRESTNVVDVCTERNEVIVRLRGGGGAGASGKNQSLGGSAAASANAARPQGNAAGSNSSKVFRFDGVCPVSTSQEQ
P52732_KIF11_Humans            ASQPNSSAKKKEEKGKNIQVVVRCRPFNLAERKASAHS-IVECDPVRKEVSVRTGG------------------------LADKS---SRKTYTFDMVFGASTKQID
P28742_KIP1_Yeast              TLTPPTCNNGAATSDSNIHVYVRCRSRNKREIEEKSSVVISTLGPQGKEIILSNG--------------------------SHQSYSSSKKTYQFDQVFGAESDQET

                               110 120 130 140 150 160 170 180 190 200 210
cgd6_4210_C_parvum             LFKQYVVPIVDEVLLGFNCTIFAYGQTGTGKTYTMEGDM--------------------KEYLESSNLELTEHAGIIPRAVQLIFERLESQYT--------------
CMU_020550_C_muris             LFKQYIVPIVDEALLGFNCTIFAYGQTGTGKTYTMEGDM--------------------KEYLEISNISLSDHAGIIPRAVQLIFERLESQYT--------------
PF3D7_0317500_P_falciparum     LFNNYIYQIVDEVLQGFNCTLFCYGQTGTGKTYTMEGKILEHLKQYDNNKKVDLNESINSDISYCYELCENEDTGLIFRVTKRIFDILNKRKEEKIRHFKGKKNIFD
TGME49_297110_T_gondii         FFEQHAQPLVDEVLQGFNCTIFAYGQTGTGKTFTIEGAPRG---------------GGESIYSANDEISENADCGLVGRSVQRIFSTLRGEHVAK------------
P52732_KIF11_Humans            VYRSVVCPILDEVIMGYNCTIFAYGQTGTGKTFTMEGER--------------------SPNEE-YTWEEDPLAGIIPRTLHQIFEKLTDNGT--------------
P28742_KIP1_Yeast              VFNATAKNYIKEMLHGYNCTIFAYGQTGTGKTYTMSGDINI-----------------LGDVQSTDNLLLGEHAGIIPRVLVDLFKELSSLNK--------------

                               220 230 240 250 260 270 280 290 300 310 320
cgd6_4210_C_parvum             -------------------------------------------------------------------------------------------EYGVRVSYLEIYNEEL
CMU_020550_C_muris             -------------------------------------------------------------------------------------------EYGVRVSYLEIYNEEL
PF3D7_0317500_P_falciparum     EKDNEHINVINKETNGNNNNIINNNYNNLLCNKINIDKNDIYNNLKINKEPNNLLVNEKSRDITCDVVQKCSVDKNRNNDMYQTFDDKNMYDFNIKISYLEIYNEEL
TGME49_297110_T_gondii         -------------------------------------------------------------------------------------------DYTVTCSFLEIYNEEL
P52732_KIF11_Humans            -------------------------------------------------------------------------------------------EFSVKVSLLEIYNEEL
P28742_KIP1_Yeast              -------------------------------------------------------------------------------------------EYSVKISFLELYNENL

                               330 340 350 360 370 380 390 400 410 420
cgd6_4210_C_parvum             SDLLS---------------------------------DEKLNLRIYDDIAGK-----------------------RGLNVDRLEEIPVNKAQDILNILSTAVRKRR
CMU_020550_C_muris             SDLLS---------------------------------DEKVSLKIYDDTSGK-----------------------RGLNVDKLEEMPVNKAQDIFNILSTAVRKRR
PF3D7_0317500_P_falciparum     CDLLSST-------------------------------NENMKLRIYEDSNNKS----------------------KGLNVDKLEEKSINSFEEIYYIICSAIKKRR
TGME49_297110_T_gondii         IDLFAPAPASAGGSSCPSTQPSAACSDSNDSQASQSGALSTPRLRIYEDHVSSTTLDAAGAGGSVSMAARGPQQTRGVVRVEGLEEKEVKSEREVLALLRAAAPRRS
P52732_KIF11_Humans            FDLLNPS------------------------------SDVSERLQMFDDPRNK-----------------------RGVIIKGLEEITVHNKDEVYQILEKGAAKRT
P28742_KIP1_Yeast              KDLLSDSEDDD-----------------------PAVNDPKRQIRIFDNNNNN-----------------------SSIMVKGMQEIFINSAHEGLNLLMQGSLKRK

                               430 440 450 460 470 480 490 500 510 520 530
cgd6_4210_C_parvum             TAETLLNKSSSRSHCIFTITIHTKETNID------------------------------GEDVLKVGKLNLVDLAGSENIQRSGANAV-KDRAKEAGMINQSLLTLG
CMU_020550_C_muris             TAETLLNKCSSRSHCIFTITIHTKETNIE------------------------------GEDVLKVGKLNLVDLAGSENIQRSGANAI-KDRAKEAGMINQSLLTLG
PF3D7_0317500_P_falciparum     TAETAYNKKSSRSHSIFTITLIIKDINNV------------------------------GESITKIGKLNLVDLAGSENALKSSYGSL-KIRQQESCNINQSLLTLG
TGME49_297110_T_gondii         FAASSANAHSSRSHTIFSISVCIRDTANALSDEGGDREGAKSRKGGDPAFRVSTVSAPVEEEVVRVGKLNLVDLAGSENIYKSWGATAEEKRRREALTINKSLLTLG
P52732_KIF11_Humans            TAATLMNAYSSRSHSVFSVTIHMKETTID------------------------------GEELVKIGKLNLVDLAGSENIGRSGA--V-DKRAREAGNINQSLLTLG
P28742_KIP1_Yeast              VAATKCNDLSSRSHTVFTITTNIVEQDSKDHGQ--------------------------NKNFVKIGKLNLVDLAGSENINRSGAE---NKRAQEAGLINKSLLTLG

                               540 550 560 570 580 590 600 610 620 630
cgd6_4210_C_parvum             RVINALVEHSSYVPYRDSKLTRLLQDSLGGRTKTCIIATITASSIYLEETLNTLDYAHRAKNIKNMPVVNQKMTKKVMIREMNCEIEKLKQELQCNREKNGV
CMU_020550_C_muris             RVINALVDHSSYVPYRDSKLTRLLQDSLGGRTKTCIIATVTSSSLYLEETLNTLDYAHRAKNIKNMPVVNQKMTKKVMIREMNCEIERLKFELQANREKNGV
PF3D7_0317500_P_falciparum     RVINSLIENSSYIPYRDSKLTRLLQDSLGGKTKTFIVATISPSSLCIDETLSTLDYVFRAKNIKNRPEINIKTTKQLKIKDLNNEIEKLRNALNLSREKRGV
TGME49_297110_T_gondii         RCINALVDRASYVPYRDSKLTRLLQDSLGGCTKTCLIATISPAENVVEETINTLDYAYRAKSIQNLPVKTLRHSKSLLLSSLLQENQQLKLLLCSQRERDGV
P52732_KIF11_Humans            RVITALVERTPHVPYRESKLTRILQDSLGGRTRTSIIATISPASLNLEETLSTLEYAHRAKNILNKPEVNQKLTKKALIKEYTEEIERLKRDLAAAREKNGV
P28742_KIP1_Yeast              RVINALVDHSNHIPYRESKLTRLLQDSLGGMTKTCIIATISPAKISMEETASTLEYATRAKSIKNTPQVNQSLSKDTCLKDYIQEIEKLRNDLKNSRNKQGI
```

Fig. S9. Molecular docking showing the binding of SB743921 (purple) to Cpkin5 motor domain (light green) that was superimposed with a human kinesin-5/Eg5 model (4BXN).

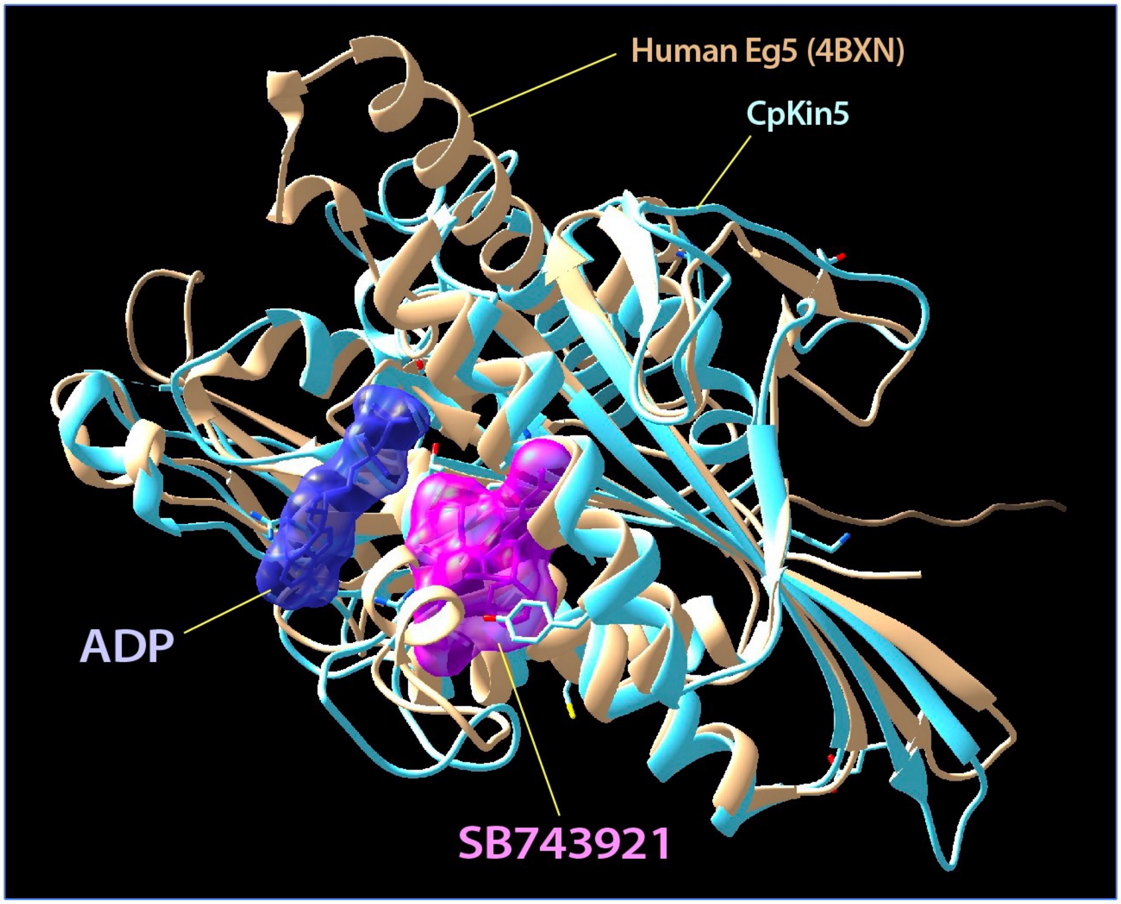

Supplement: Figures S1 to S9 — Additional immunostaining images, sequence alignments, and molecular docking models. [file mbio.03158-23-s0001.pdf]
