# Supplementary material for: Requirement of microtubules for secretion of a micronemal protein CpTSP4 in the invasive stage of the apicomplexan Cryptosporidium parvum
Source: mBio. 2024 Jan 24;15(2):e03158-23. doi: 10.1128/mbio.03158-23 (PMC10865969; doi:10.1128/mbio.03158-23)
Supplement: Table S1 — List of TSP/TRAP family proteins in Cryptosporidium parvum. [file mbio.03158-23-s0002.pdf]

Table S1. List of TSP/TRAP family proteins in *Cryptosporidium parvum*.

Name	Gene ID	GenBank #	Length (aa)	CryptoDB Product Description	SignalP	No. TMD
CpTSP1 (TRAP-C1)	cgd1_3500	XP_628162	687	Thrombospondin related adhesive protein	Yes	1
CpTSP2 (TRAP-C2)	cgd5_3420	XP_626266	3869	TSP1/LNR (Lin-12/Notch) repeat containing protein	Yes	1
CpTSP3	cgd1_3510	XP_628163	507	PAN domain/Thrombospondin type-1 (TSP1) repeat containing protein	Yes	0
**CpTSP4**	**cgd8_150**	**XP_625479**	**488**	**Thrombospondin type-1 (TSP1) repeat-containing protein**	**Yes**	**0**
CpTSP5	cgd6_1300	XP_627501	354	Uncharacterized protein with Thrombospondin type-1 (TSP1) repeat	Yes	0
CpTSP6	cgd6_2310	XP_627588	358	Signal Peptide Uncharacterized transmembrane Protein	Yes	1
CpTSP7	cgd5_4470	XP_625608	656	Thrombospondin type-1 (TSP1) repeat/EGF-like domain containing protein	No	2
CpTSP8 (CpMIC1)	cgd6_780	XP_627453	625	Thrombospondin type-1 (TSP1) repeat/EGF-like domain containing protein	No	2
CpTSP9	cgd6_800	XP_627454	457	Thrombospondin type-1 (TSP1) repeat/EGF-like domain containing protein	Yes	1
CpTSP10	cgd2_3080	XP_626502	391	CpTSP10 protein/Kringle domain containing protein	Yes	1
CpTSP11	cgd6_1660	XP_627532	1126	Uncharacterized protein with Thrombospondin type-1 (TSP1) repeat	Yes	0
CpTSP12	cgd8_540	XP_625515	825	Thrombospondin type-1 (TSP1) repeat containing protein	Yes	0

Note: **Bold blue font (CpTSP4)** indicates the protein under investigation in this study. Signal peptide (SignalP) and the number of transmembrane domains (No. TMD) were predicted by Phobius.
