# Supplementary material for: Requirement of microtubules for secretion of a micronemal protein CpTSP4 in the invasive stage of the apicomplexan Cryptosporidium parvum
Source: mBio. 2024 Jan 24;15(2):e03158-23. doi: 10.1128/mbio.03158-23 (PMC10865969; doi:10.1128/mbio.03158-23)
Supplement: Table S3 — Similarity and identity scores between kinesin-5 motor domains from selected species. [file mbio.03158-23-s0004.pdf]

Table S3. Similarity and identity scores between kinesin-5 motor domains from selected species.

**Species (gene ID or GenBank #)**	***C. parvum***	***C. muris***	***P. falciparum***	***T. gondii***	**Humans**	**Yeast**
	**Identity scores based on Gonnet scoring matrix**					
C. parvum (cgd6_4210)	100	87.3	39.7	35.8	55.2	47.1
C. muris (CMU_020550)	95.3	100	39.1	36.8	54	45.9
P. falciparum (PF3D7_0317500)	54.4	54.1	100	28.1	33.8	32.7
T. gondii (TGME49_297110)	49.9	49.7	41	100	34.5	32.8
Humans (P52732)	73.3	72.8	48.3	49.9	100	47.5
Yeast (P28742)	63.9	62.7	45.8	47.8	64.7	100.0
	**Similarity scores based on Gonnet scoring matrix**					
