# Supplementary material for: Requirement of microtubules for secretion of a micronemal protein CpTSP4 in the invasive stage of the apicomplexan Cryptosporidium parvum
Source: mBio. 2024 Jan 24;15(2):e03158-23. doi: 10.1128/mbio.03158-23 (PMC10865969; doi:10.1128/mbio.03158-23)
Supplement: Table S2 — Similarity and identity scores of the motor domains between the seven C. parvum kinesin superfamily proteins. [file mbio.03158-23-s0003.pdf]

Table S2. Similarity and identity scores of the motor domains between the seven *C. parvum* kinesin superfamily proteins.

Kinesin type	GenBank #	CryptoDB Gene ID	CpKin5	CpKin1	CpKinX6	CpKin14	CpKin13	CpKin-like	CpKin8X
			Identity scores based on Gonnet scoring matrix						
**CpKin5**	XP_001388347	cgd6_4210	100.0	27.6	32.8	18.3	28.2	19.5	23.1
**CpKin1**	XP_001388331	cgd6_2790	42.4	100.0	26.8	25.3	21.2	19.8	20.4
**CpKinX6**	XP_001388319	cgd6_1920	46.3	38.7	100.0	18.0	23.1	21.4	21.1
**CpKin14**	XP_001388114	cgd3_2590	31.3	38.3	31.4	100.0	15.1	16.7	15.1
**CpKin13**	XP_627225	cgd8_3300	45.0	37.0	38.0	28.8	100.0	17.7	25.7
**CpKin-like**	XP_001388318	cgd6_1900	35.0	32.8	36.1	28.1	34.9	100.0	15.9
**CpKin8X**	XP_001388120	cgd3_3090	37.5	34.3	35.3	27.3	40.0	31.6	100.0
			Similarity scores based on Gonnet scoring matrix						
